# Dnmt1/Tet2-mediated changes in Cmip methylation regulate the development of nonalcoholic fatty liver disease by controlling the Gbp2-Pparγ-CD36 axis

**DOI:** 10.1038/s12276-022-00919-5

**Published:** 2023-01-06

**Authors:** Jangho Lee, Ji-Hye Song, Jae-Ho Park, Min-Yu Chung, Seung-Hyun Lee, Sae-Bom Jeon, So Hee Park, Jin-Taek Hwang, Hyo-Kyoung Choi

**Affiliations:** 1 Division of Food Functionality Research, Korea Food Research Institute, Jeollabuk-do, 55365 Republic of Korea; 2 Department of Food and Nutrition, Gangseo Univ., Seoul, 07661 Republic of Korea; 3 Department of Biochemistry and Molecular Biology, College of Medicine, Yonsei Univ., Seoul, 03722 Republic of Korea; 4 Division of Cardiology, Department of Medicine, Johns Hopkins University, MD 21205 Baltimore, USA; 5 Department of Food Biotechnology, Korea University of Science & Technology, Daejeon, 34113 Republic of Korea

**Keywords:** Cell signalling, Mechanisms of disease

## Abstract

Dynamic alteration of DNA methylation leads to various human diseases, including nonalcoholic fatty liver disease (NAFLD). Although C-Maf-inducing protein (Cmip) has been reported to be associated with NAFLD, its exact underlying mechanism remains unclear. Here, we aimed to elucidate this mechanism in NAFLD in vitro and in vivo. We first identified alterations in the methylation status of the *Cmip* intron 1 region in mouse liver tissues with high-fat high-sucrose diet-induced NAFLD. Knockdown of DNA methyltransferase (*Dnmt*) 1 significantly increased Cmip expression. Chromatin immunoprecipitation assays of AML12 cells treated with oleic and palmitic acid (OPA) revealed that Dnmt1 was dissociated and that methylation of H3K27me3 was significantly decreased in the *Cmip* intron 1 region. Conversely, the knockdown of Tet methylcytosine dioxygenase 2 (*Tet2*) decreased Cmip expression. Following OPA treatment, the CCCTC-binding factor (Ctcf) was recruited, and H3K4me3 was significantly hypermethylated. Intravenous *Cmip* siRNA injection ameliorated NAFLD pathogenic features in *ob/ob* mice. Additionally, *Pparγ* and *Cd36* expression levels were dramatically decreased in the livers of *ob*/*ob* mice administered si*Cmip*, and RNA sequencing revealed that Gbp2 was involved. *Gbp2* knockdown also induced a decrease in *Pparγ* and *Cd36* expression, resulting in the abrogation of fatty acid uptake into cells. Our data demonstrate that Cmip and Gbp2 expression levels are enhanced in human liver tissues bearing NAFLD features. We also show that Dnmt1–Trt2/Ctcf-mediated reversible modulation of *Cmip* methylation regulates the Gbp2–Pparγ–Cd36 signaling pathway, indicating the potential of *Cmip* as a novel therapeutic target for NAFLD.

## Liver disease: Epigenetic modification and nonalcoholic fatty liver

Changes in the chemical modifications of a gene involved in immune cell development and associated with the metabolic disease lead to the activation of a signaling pathway that promotes the onset and progression of nonalcoholic fatty liver disease (NAFLD). This metabolic disorder can lead to cirrhosis and liver failure. Several studies have tentatively linked C-Maf-inducing protein (Cmip) to NAFLD. Researchers led by Jin-Taek Hwang and Hyo-Kyoung Choi of the Korea Food Research Institute, Wanju-gun, South Korea, have now demonstrated that this protein is elevated in NAFLD through the action of enzymes that remove inhibitory chemical modifications associated with the gene encoding Cmip. Cmip, in turn, triggers the activation of numerous other proteins associated with NAFLD pathology. The authors also show that Cmip inhibition can alleviate this liver condition in animal models, highlighting a potential therapeutic approach.

## Introduction

Nonalcoholic fatty liver disease (NAFLD), the most common cause of chronic liver diseases worldwide, is a spectrum of liver disorders that occur in the context of metabolic disorders such as obesity and type 2 diabetes mellitus^1,2^. This disease encompasses a wide range of liver conditions ranging from simple hepatic steatosis to nonalcoholic steatohepatitis (NASH)^3^. Genetic and epigenetic factors are involved in the pathogenesis of NAFLD, and the underlying molecular mechanisms are being studied to discover biomarkers for the treatment of NAFLD. Recent studies have particularly focused on epigenetic mechanisms such as DNA methylation, which is modulated by environmental factors and lifestyle^4,5^.

DNA methylation is a crucial epigenetic modification of the genome that is associated with the control of many cellular processes^6^. The levels and patterns of DNA methylation occurring in CpG motifs are regulated by both DNA methyltransferases (Dmnt1, Dnmt3a, and Dnmt3b) and hydroxymethylases, including the ten-eleven translocation (TET) family of dioxygenases (Tet1, Tet2, and Tet3)^7^. Dysregulation of DNA methylation is implicated in the pathogenesis of numerous human diseases^8^, including NAFLD, the development of which may involve epigenetic mechanisms associated with “metabolic memory”^9^. Global DNA hypomethylation and abnormal DNA methylation were found in lipogenic diet-induced hepatic steatosis and NASH development in a mouse model^10–12^, and altered DNA methylation is involved in hepatic steatosis^10^ and NAFLD-related disorders^3,13^. A comprehensive, genome-wide methylation study found extensive DNA methylation changes in more than 100 genes associated with lipid and glucose metabolism^14^. Furthermore, some genes encoding key enzymes associated with metabolic disorders, including *Igf1* (insulin-like growth factor 1), *Igfbp2* (IGF binding protein 2), *Acly* (ATP citrate lyase), and *PC* (pyruvate carboxylase), have been observed to have a specific methylation signature that distinguishes NAFLD from other liver disorders^1^.

C-Maf-inducing protein (Cmip), which was first identified in the human brain^15^, is involved in multiple signaling pathways related to nuclear factor-κB^16^ and T-helper 2^17^ signaling. Recently, evidence suggesting that Cmip is involved in metabolic diseases has emerged. For example, a study revealed that polymorphism of *Cmip* is associated with T2DM and obesity risk^18^. Additionally, *Cmip* activates arginase-1, a known risk factor for cardiovascular disorders^19^. However, the mechanisms underlying *Cmip*-mediated metabolic diseases and the regulation of Cmip expression remain unclear.

In the present study, we aimed to evaluate and demonstrate the functional importance of *Cmip* in the development of NAFLD. We evaluated the change in mRNA expression in mice fed a high-fat high-sucrose (HFHS) diet as we aimed to identify a potential and reliable biological target for the prevention and treatment of NAFLD.

## Materials and methods

### Reagents and antibodies

Oleic acid, palmitic acid, SGI-1027, and BSA (essentially fatty acid-free) were purchased from Sigma‒Aldrich (St. Louis, MO, USA). Dimethyl sulfoxide (DMSO) was purchased from Duchefa Biochemie (BV, Haarlem, The Netherlands).

### Cell culture

AML12 cells were purchased from the American Type Culture Collection (Manassas, VA, USA). The cells were cultured in Dulbecco’s modified Eagle’s medium/F-12 (Gibco, Grand Island, NY, USA) supplemented with 10% fetal bovine serum (Welgene, Daegu, Korea), antibiotic/antimycotic solution (Welgene), insulin-transferrin-selenium solution (10 µg/mL insulin, 5.5 µg/mL transferrin, and 5 ng/mL selenium; Invitrogen, Carlsbad, CA, USA), and 40 ng/mL dexamethasone (Sigma‒Aldrich). To establish a NAFLD model using AML12 cells, we used a nonfat BSA-conjugated combination of oleic acid and palmitic acid (OPA) at a ratio of 4:1.

### Bisulfite amplicon sequencing (BSAS)

One microgram of genomic DNA was bisulfite-converted using EZ DNA Methylation (Zymo Research, Orange, CA, USA) according to the manufacturer’s protocol. The library was prepared with an Illumina TruSeq Nano DNA Sample Prep Kit (Illumina, San Diego, CA, USA) according to the manufacturer’s instructions. Libraries were quantified by quantitative PCR using a CFX96 Real-Time System (Bio-Rad, Hercules, CA, USA). After normalization, sequencing of the prepared library was conducted on the MiSeq system (Illumina) with 300-bp paired-end reads.

### Immunoblotting

Cells were washed with cold PBS and collected. Cell extracts were prepared using RIPA buffer (Elpis, Daejeon, Republic of Korea) containing protease and phosphatase inhibitors (Roche, Basel, Switzerland) and incubated on ice for 30 min. The lysates were centrifuged at 20,000 × *g* for 20 min at 4 °C. The cell lysates were separated on SDS‒PAGE gels and then transferred to nitrocellulose membranes. The membranes were blocked in 5% (w/v) nonfat Difco™ skim milk solution in 1× PBST for 1 h. The blocked membranes were incubated overnight at 4 °C with the indicated primary antibodies (Supplementary Table 1). The membranes were then washed with 1× PBST, incubated with the appropriate secondary anti-rabbit or anti-mouse horseradish peroxidase-conjugated antibody (Thermo Scientific, Rockford, IL, USA) for 1 h, and visualized using an imaging system (Vilber Lourmat, ZAC de Lamirault, France) with an enhanced chemiluminescence detection reagent (Thermo Scientific).

### qRT‒PCR

Cells were seeded in six-well plates at 2 × 10^5^ cells/well. After reaching ~70% confluence, total RNA was isolated using TRIzol reagent (Invitrogen). qRT‒PCR was performed using an I Cycler iQ system (Bio-Rad) with SYBR Green PCR master mix (Thermo Fisher Scientific, Waltham, MA, USA). PCR amplification was carried out in triplicate using the primers listed in Supplementary Table 2. mRNA levels were normalized to those of β-actin mRNA, and relative expression levels were calculated using the comparative 2^−ΔΔCT^ method^20^.

### ChIP assays

Cells (2 × 10^8^) were seeded in 15-mm dishes with or without OPA at the indicated concentrations for 24 h until ~80% confluence was reached. Cells were initially fixed with PBS containing 1% formaldehyde for 10 min and washed three times with cold PBS. Then, cross-linking was stopped by adding 125 mM glycine for 5 min at 24–26 °C. Subsequent processes were carried out according to the manufacturer’s protocol for the Pierce Agarose ChIP Kit (Thermo Fisher Scientific) using the indicated antibodies essentially as described (Supplementary Table 1) but without SDS in any buffers. The primers used to amplify the target region in chromatin are listed in Supplementary Table 2. All reactions were normalized relative to input activities and are presented as the mean ± standard deviation (SD) of three independent experiments.

### siRNA transfection

Cells were seeded in six-well plates at 2 × 10^5^ cells/well. After reaching approximately 70% confluence, the cells were transfected with the indicated siRNA (Genolution, Seoul, Republic of Korea) using Lipofectamine RNAiMAX reagent (Thermo Fisher Scientific) for 24, 48, or 72 h. The sequences of siRNA duplexes are shown in Supplementary Table 3. After transfection, the cells were subjected to further experiments (qRT‒PCR or immunoblotting).

### Measurement of cellular free fatty acid uptake

Fatty acid uptake was determined using 4,4-difluoro-5,7-dimethyl-4-bora-3α,4α-diaza-s-indacene-3-hexadecanoic acid (Molecular Probes, Eugene, OR) in AML12 cells as described in a previous study^19^. The fluorescence intensity at 488/514 nm was measured by a SpectraMax M_2_ microplate reader (Molecular Devices, Sunnyvale, CA, USA).

### Gene Ontology (GO) enrichment analysis

Two public datasets, listed in Supplementary Table 4, were refined and analyzed using the bioinformatics tools in the integrative Library of Integrated Network-Based Cellular Signatures genomics data portal (http://www.ilincs.org). The signatures were defined by the following variable groupings: ID, gdsGDS6248; samples, normal diet (ND; *n* = 3; sample ID, GSM994696–GSM994798) vs. high-fat diet (HFD; *n* = 3; sample ID, GSM994820–GSM994822); time, 6 weeks; ID, greinGSE95428; genotype, wildtype (wt); samples, ND (*n* = 4; sample ID, GSM2510587–GSM2510589) vs. HFD (*n* = 4; sample ID, GSM2510591–GSM2510594). Genes with *p* values < 0.01 were selected in each signature and subjected to GO enrichment analysis using DAVID Bioinformatics Resource 6.8^21^.

### Animal experiments

Animal experiments using high-fat high-sucrose (HFHS) diet-fed mice were conducted according to the Guide for the Korea Food Research Institutional Animal Care and Use of Committee (KFRI-M-15012). The animal experiments using C57BL/6J-*wt* (wt) and C57BL/6J*-ob/ob* (*ob/ob*) mice were performed in accordance with the National Institute of Health Guide for the Care and Use of Laboratory Animals and were approved by the Ulsan University Guide for the Animals Care and Use Committee (2019-02-361). Specific information on animal experiments is specified in the Supplementary Information for animal experiments.

### Blood biochemical analysis

Serum isolated from blood samples collected via abdominal heart puncture was used for enzymatic measurements of the levels of aspartate transaminase, alanine aminotransferase, alkaline phosphatase, triglycerides (TG), total cholesterol, low-density lipoprotein (LDL) cholesterol, and high-density lipoprotein (HDL) cholesterol. All measurements were performed using commercial kits in accordance with the manufacturer’s protocols (Asan Pharm, Seoul, Korea).

### H&E staining

Liver and fat specimens were fixed in 4% buffered formalin, embedded in paraffin, and cut into 4–5-μm-thick sections, which were stained with H&E. Lipid accumulation in livers was visually assessed using an Eclipse 80i microscope (Nikon Instruments, Inc., Melville, NY, USA).

### Immunohistochemistry, interpretation, and scoring

Tissue microarray slides (LV1201b) containing 118 liver tissues were purchased from US Biomax, Inc. (Rockville, MD, USA). Immunohistochemistry was performed using a Klear Mouse HRP with a DAB kit (GBI Lab, Bothell, WA, USA) according to the manufacturer’s protocol. Briefly, for antigen retrieval, slides were heated in citrate butter (pH 6.0) for 30 min and exposed to Protein Block Serum-Free blocking solution (Dako, Carpinteria, CA, USA) to block nonspecific background staining. The primary antibody, CMIP (1:100), or guanylate-binding protein 2 (GBP2; 1:200), was incubated overnight at 4 °C and visualized using 3,3’-diaminobenzidine. Two blinded pathologists interpreted the expression of CMIP and GBP2 in 14 normal tissues and 13 tissues showing clinical signs of NAFLD without cancer after H&E staining. The degree of staining in normal hepatocytes was set to 0, and that in hepatocytes from NAFLD tissues was scored relative to 0. The difference in the expression levels of Cmip and Gbp2 was quantified according to the clinical reading guide.

### Statistical analysis

Data were analyzed using Student’s *t*-test or one-way analysis of variance with Tukey’s multiple comparison test or Pearson’s correlation analysis, and values are expressed as the mean ± SD or Pearson’s correlation coefficient (*R)*, respectively. Statistical analyses were conducted using GraphPad Prism (version 7.04, GraphPad Software, Inc., San Diego, CA, USA). Differences were considered statistically significant at *p* < 0.05.

## Results

### Hypomethylation in *Cmip* intron 1 enhances *Cmip* expression in obesity-induced NAFLD

**Fig. 1:**
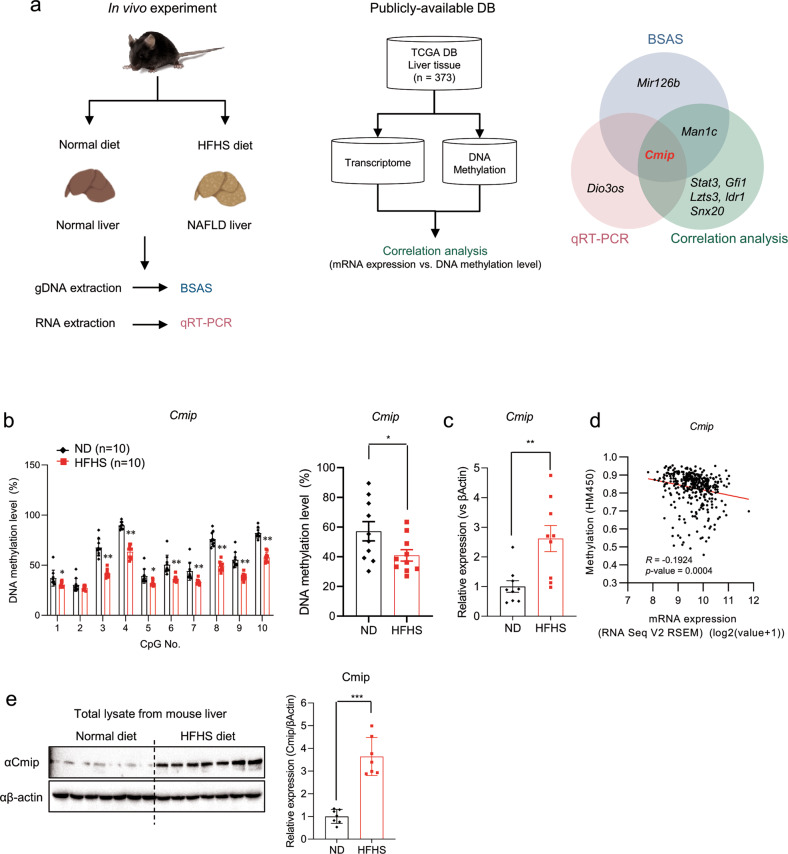
Hypomethylation of the *Cmip* intron 1 region increases its mRNA and protein expression levels in high-fat high-sucrose diet-supplemented mice. **a** Experimental design to identify genes that regulate nonalcoholic fatty liver disease (NAFLD) by DNA methylation. C57BL/6N mice were fed an HFHS diet or a normal diet and were sacrificed 12 weeks later. gDNA or total RNA was extracted from liver tissues and used for RRBS or qRT‒PCR experiments to measure DNA methylation or mRNA expression levels, respectively (left panel). The correlation between the transcriptome and DNA methylome of human liver tissues was analyzed using TCGA DB (liver tissue, *n* = 373, RNA-seq and HM450K methylation data from GDAC Firehose) (middle panel). Venn diagram shows shared or unique genes in each of the three analyses (right panel). BSAS bisulfite amplicon sequencing, qRT‒PCR quantitative reverse transcription PCR, gDNA guide DNA, HFHS high-fat high-sucrose. **b** Schematic of the Cmip methylation status in the liver tissues of mice fed either a normal diet (ND) or an HFHS diet. The methylation status of CpG islands of the *Cmip* intron 1 region from +117,326,061 to 117,325,350 was measured by BSAS in both the ND- and HFHS diet-supplemented groups. The methylation level of each site was calculated (left panel). The total methylation level between the ND- and HFHS diet-fed groups was compared (right panel). The values presented are the means ± SEs of ten mice per group. *p < 0.05, **p < 0.01, and ***p < 0.001; Student’s *t*-test. **c** *Cmip* expression in the livers of mice fed either the ND or HFHS diet. The values presented are the means ± SEs of ten independent mice per group. ***p < 0.001; Student’s *t*-test. **d** Correlation between *CMIP* mRNA expression and DNA methylation levels in human liver tissues (*n* = 373). Pearson’s correlation analysis was performed. **e** The protein contents of Cmip in the liver tissues of mice fed either the ND or HFHS diet. *Cmip* expression was measured in ND and HFHS diet-fed mice. The protein expression of Cmip was measured by western blotting. The liver lysates were immunoblotted with the indicated antibodies (left panel). The intensities of the protein bands obtained from the western blot assay were quantified with ImageJ (right panel) and normalized with respect to the intensity of β-actin. The relative fold intensity was calculated as the sum of the normalized intensities from both β-actin and Cmip. ***p < 0.001; Student’s *t*-test.

Through whole genome reduced-presentation bisulfite sequencing (RRBS), we identified 13 genes, including *Cmip*, that showed more than 20% decreased or increased DNA methylation patterns in the livers of HFHS diet-supplemented C57BL/6N mice relative to ND-supplemented mice for 12 weeks (data not shown). We identified candidate genes that regulate NAFLD by combining the results of the BSAS and qRT‒PCR of the mouse livers and the correlation analysis between the transcriptome and DNA methylome of human liver tissues. Correlation analysis was performed using the RNA-seq and HM450k data of human liver tissues from The Cancer Genome Atlas (TCGA) database (*n* = 373), and genes showing a negative correlation were selected (Fig. 1a). BSAS analysis of the 13 candidate genes revealed that *Cmip*, *Mir126b*, and *Man1c1* showed significant decreases in DNA methylation levels in at least three CpG sites in mice fed the HFHS diet, and *Pex26* showed an increase in DNA methylation levels (Fig. 1b, Supplementary Fig. 1a, and Supplementary Table 5). Notably, we found that nine of ten CpG sites in the *Cmip* intron 1 region (Supplementary Fig. 2) were hypomethylated in the livers of HFHS diet-supplemented mice, and the total methylation level of the CpG sites decreased by approximately 30% relative to that in ND-supplemented mice (*p* < 0.05, Fig. 1b).

Analysis of the mRNA expression of the 13 genes showed that *Cmip* and *Dio3os* expression levels were significantly increased, and those of *Alox5ap* and *Pex26* were significantly decreased in mice fed an HFHS diet (*Gfi1*, *Snx20*, *Mir126b*, *Ripor3*, and *Ildr1* expression levels were not detected; Fig. 1c, Supplementary Fig. 1b). Additionally, using the cBioPortal platform (https://www.cbioportal.org), we performed correlation analysis of gene expression and DNA methylation levels using human liver tissue transcriptome and DNA methylation data (*n* = 373) from publicly available databases. Seven genes, including *CMIP*, showed significant negative correlations with DNA methylation (*p* < 0.01, Fig. 1d and Supplementary Fig. 3). Therefore, *Cmip* was chosen as a candidate gene that regulates obesity-induced NAFLD. Additionally, the protein expression of hepatic Cmip was significantly increased in HFHS diet-fed mice compared with normal diet (ND)-fed mice (*p* < 0.05, Fig. 1e). In addition, *Cmip* expression levels in mice fed either an ND or a high-fat diet (HFD) were analyzed using data from the public transcriptome datasets GDS6248 and GSE5428 (Supplementary Table 4), and it was found that *Cmip* expression was significantly higher in the HFD-fed mouse groups (Supplementary Fig. 4). In obesity-induced NAFLD, methylation of the *Cmip* intron 1 region was reduced, and *Cmip* mRNA and protein expression levels were increased. Taken together, HFHS diet-induced hypomethylation in the Cmip intron 1 region increases its expression.

### Ctcf and Tet2 are responsible for the regulation of Cmip expression

**Fig. 2:**
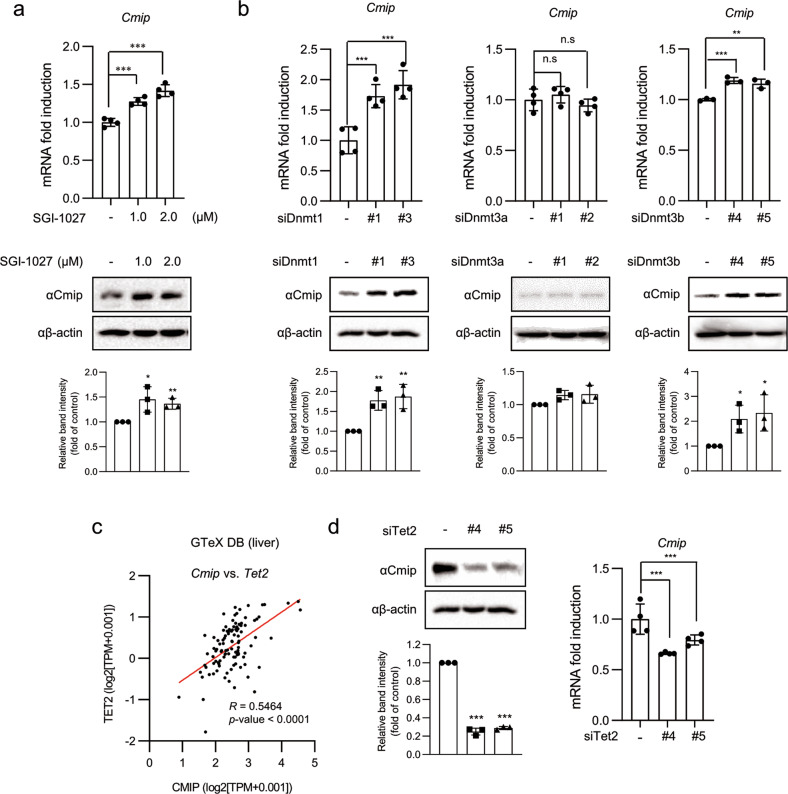
Dnmt1 and Tet2 reversely regulate Cmip expression. **a** The effect of the DNMT inhibitor (DNMTi) SGI-1027 on Cmip expression. Cmip expression levels were increased following SGI-1027 exposure. Cells were treated with SGI-1027 at the indicated concentrations for 24 h. mRNA expression of *Cmip* was measured by qRT‒PCR. The values presented are the means ± SDs of three independent experiments (upper panel). The level of Cmip protein was detected by western blot analysis, and the intensities of the protein bands were quantified using FusionCapt Advance Solo 7 software (lower panel). *p < 0.05, **p < 0.01, and ***p < 0.001; one-way ANOVA followed by Tukey’s multiple test. **b** The effect of *Dnmt* knockdown on Cmip expression in AML12 cells. The values presented are the means ± SDs of three independent experiments (upper panels). Cmip protein levels were measured by western blotting, and the intensities of the protein bands were quantified using FusionCapt Advance Solo 7 software (lower panels). n.s not significant (*p* < 0.05), *p < 0.05, **p < 0.01, and ***p < 0.001; Student’s *t*-test. **c** The correlation of the relative influence between Cmip and Tet2 expression levels in human liver tissues (110 samples) from the Genotype-Tissue Expression (GTeX) database. The correlation between the expression of *Cmip* and *Tet2* was expressed as the Pearson correlation coefficient (*R*). Pearson’s correlation analysis was performed. **d** The effect of *Tet2* knockdown on Cmip expression in AML12 cells. The values presented are the means ± SDs of three independent experiments (right panel). Cmip protein levels were measured by western blotting, and the intensities of the protein bands were quantified using FusionCapt Advance Solo 7 software (left panel). ***p < 0.001; Student’s *t*-test.

To confirm that inhibition of Dnmt activity influences Cmip expression, AML12 cells were treated with the DNMT inhibitor SGI-1027 at the indicated concentrations. As expected, both the mRNA and protein expression levels of Cmip were significantly increased (Fig. 2a).

Next, to investigate which DNMT is involved in *Cmip* methylation, we adapted the knockdown system using siRNA. The top two siRNAs showing the highest knockdown effect against five target sequences per gene (Supplementary Fig. 5 and Fig. 2b) were transiently transfected into AML12 cells. *Cmip* expression was significantly increased in cells with Dnmt1 knockdown (Fig. 2b, upper left panel), and Dnmt3b knockdown also facilitated *Cmip* expression, albeit to a lesser extent (Fig. 2b, upper right panel). However, the expression of *Cmip* was not affected by Dnmt3a (Fig. 2b, upper middle panel); its protein expression level also showed the same pattern as that of its mRNA expression level (Fig. 2b, lower panel).

To determine whether DNA hydroxymethylase is implicated in the reversible Cmip control of Dnmt1, the strength of the association between *Cmip* and the mRNA expression of enzymes responsible for DNA hydroxymethylation was analyzed using liver tissue data from the Genotype-Tissue Expression (GTeX) and TCGA databases. Gene expression of *Tet2* showed a high correlation with *Cmip* expression in both databases (*R* > 0.5) (Fig. 2c and Supplementary Table 6), suggesting a strong association between the expression of these genes. To confirm that *Tet2* is involved in *Cmip* expression, the mRNA and protein levels of Cmip were measured after two sets (#4 and #5) of *Tet2* siRNA transfection in AML12 cells (Fig. 2d). Gene expression of other Tet hydroxylase family members, namely, Tet1 and Tet3, also showed a high correlation with Cmip expression in the TCGA database (*R* = 0.44 and 0.11, respectively; Supplementary Table 6); however, knockdown of *Tet1* or *Tet3* did not suppress Cmip mRNA expression in AML12 cells (Supplementary Fig. 6). Altogether, it is thought that Cmip expression is reversibly regulated by Dnmt1 and Tet2.

### Dnmt1 and Tet/Ctcf reversibly occupy the Cmip intron 1 region

**Fig. 3:**
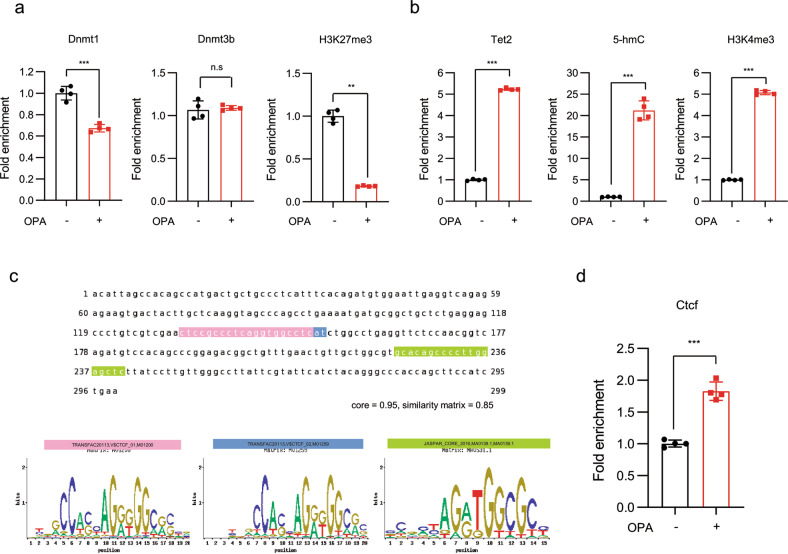
Dnmt1 and Tet2/Ctcf reversibly occupy the CpG site in the *Cmip* intron 1 region. **a** Dnmt1 occupancy and H3K27me3 methylation status in the *Cmip* intron 1 region in AML12 cells. The values presented are the means ± SDs of three independent experiments. *p < 0.05, ***p < 0.001, and n.s. nonsignificant; Student’s *t*-test. **b** Tet2, 5-hmC occupancy, and H3K4me3 methylation status in the *Cmip* intron 1 region in AML12 cells were measured using chromatin immunoprecipitation (ChIP) assays. The values presented are the means ± SDs of three independent experiments. *p < 0.05 and ***p < 0.001; Student’s *t*-test. **c** Ctcf binding conserved sequences in the *Cmip* intron 1 region. The binding probability of Ctcf to the *Cmip* intron region (chr8; 117,325,151–117,325,330) was predicted by comparing the Ctcf conserved binding motif (M01200 and M01259 from TRANSFAC; MA0139.1 from JASPAR) and the sequence of the *Cmip* intron 1 region using the ConTra v3 web server (http://bioit2.irc.ugent.be/contra/v3). **d** Ctcf occupancy in the *Cmip* intron 1 region, measured using ChIP assays. The values presented are the means ± SDs of three independent experiments. *p < 0.05 and **p < 0.01; Student’s *t*-test.

We further investigated the molecular events among DNMTs and the *Cmip* intron 1 region in vitro using ChIP assays. As shown in Fig. 3a, following OPA treatment, Dnmt1 dissociated from the methylated *Cmip* intron 1 region (Fig. 3a, left panel). Accordingly, the methylation level of H3K27me3, a gene repression marker, was significantly decreased in that region (Fig. 3a, right panel). Additionally, in the murine liver, ChIP-seq data from ChIP-Atlas showed that a high H3K27me3 ChIP signal compared to the H3K4me3 and 5-mC signals was colocalized in the Cmip intron 1 region annotating the CpG island of Cmip (Supplementary Fig. 7). However, Dnmt3b showed no change in the occupancy of the methyl-CpG region of *Cmip* (Fig. 3a, middle panel). We further confirmed that Dnmt3b directly binds to the unmethylated *Cmip* intronic region using an oligonucleotide pull-down assay in vitro (Supplementary Fig. 8). These effects consequently increased *Cmip* expression.

Next, using ChIP assay analysis, we observed the status of Tet2 occupancy, 5-hydroxymethylcytosine (5-hmC) level, and H3K4me3 methylation in the *Cmip* CpG region. Increased H3K4me3 levels in the intron regions are associated with transcription initiation and active gene status^22^. The results indicated that Tet2 hydroxymethylase was directly recruited to the region (Fig. 3b, left panel) and that 5-hmC and H3K4me3 levels were significantly increased following OPA treatment in the CpG sites (Fig. 3b, middle and right panels).

Tet2 is an active regulator of Ctcf-mediated mRNA splicing through the conversion of 5-mC to 5-hmC in an intron CpG region^23^. To evaluate the involvement of Ctcf in Cmip regulation, we first determined that the conserved sequence for Ctcf occupies the *Cmip* intron 1 region. Comparing the sequence motif with a prediction based on a position weight matrix model^24^, we observed three plausible binding sites for Ctcf that resembled a previously identified Ctcf sequence motif (Fig. 3c). As shown in Fig. 3d, the ChIP assay showed that Ctcf was recruited to the CpG region in *Cmip* intron 1 in AML12 cells following OPA treatment. These results show that Tet2 upregulated Cmip expression in a Ctcf occupancy-dependent manner.

### *Cmip* suppresses hepatic lipid accumulation through repression of the Pparγ–Cd36 axis

Based on our previous results, we revealed that *Cmip* expression was upregulated in fatty acid-induced mouse hepatocytes and HFD-fed mice and that the *Cmip* intron region was hypomethylated, along with its epigenetic regulators, Tet2, Dnmt1, and Ctcf. We further investigated whether the upregulation of Cmip is involved in fatty acid- or HFD-induced hepatic steatosis and its related downstream regulators. Thus, Gene Ontology (GO) enrichment analysis was conducted on the same publicly available datasets used to explore *Cmip* expression (Supplementary Table 4) to identify differentially expressed genes (DEGs) between the ND- and HFD-fed groups. A total of 4977 DEGs were identified, and 86 upregulated and 144 downregulated DEGs common to both groups were subjected to GO enrichment analysis. The results showed that the upregulated DEGs, including *Pex11a*, *Adrb2*, *Ccnd1*, *Cebpb*, and *Pparγ*, were enriched in the regulation of fat cell differentiation, whereas the downregulated DEGs were enriched in the sulfur amino acid metabolic process (Supplementary Fig. 9 and Supplementary Tables 7, 8).

**Fig. 4:**
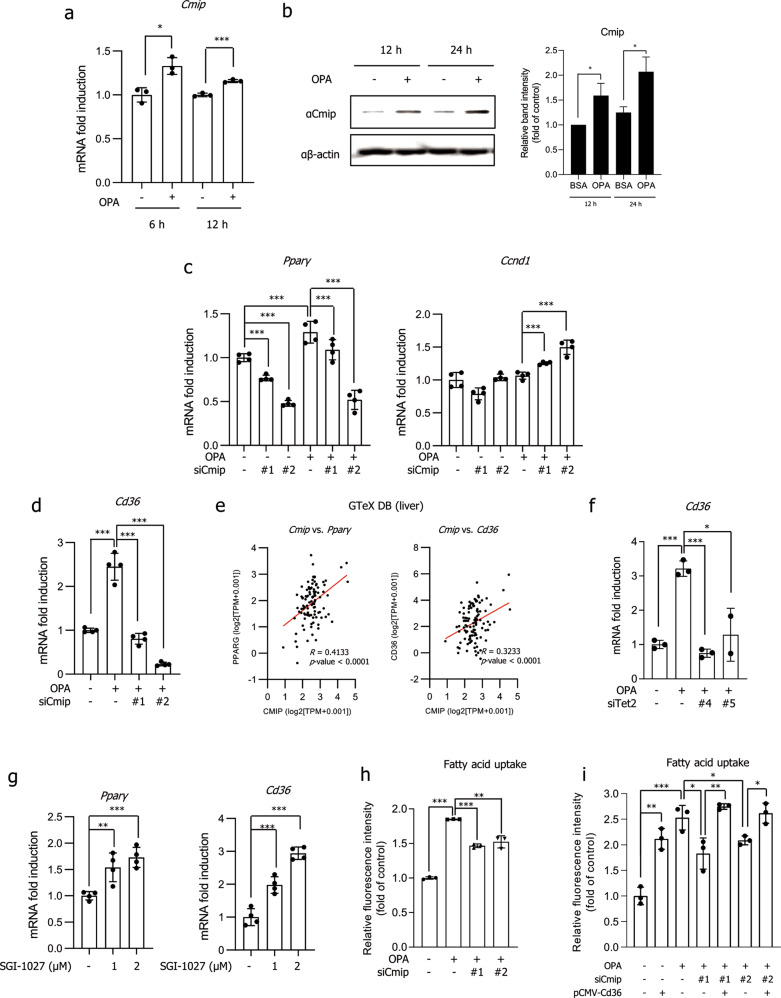
Knockdown of either *Cmip* or *Tet2* controls the Pparγ–Cd36 axis. **a**, **b** Cmip expression in AML12 cells. AML12 cells were treated with oleic acid and palmitic acid (OPA) for 6, 12, or 24 h; total RNA was extracted; and mRNA expression of *Cmip* was measured by qRT‒PCR (**a**). The values presented are the means ± SDs of three independent experiments. *p < 0.05 and **p < 0.01 (right panel); Student’s *t*-test. The protein contents of Cmip were measured 12 and 24 h after OPA treatment in AML12 cells (left panel) and quantified through normalization to the content of β-actin (**b**). *p < 0.05; Student’s *t*-test. **c**, **d** The effect of *Cmip* knockdown on *Pparγ* and *Cd36* expression. Two sets of *Cmip* siRNA were transiently transfected into AML12 cells, total RNA was extracted, and the mRNA expression levels of *Pparγ* (**c**, left panel), *Ccdn1* (**c**, right panel), and *Cd36* (**d**) were measured by qRT‒PCR. The values presented are the means ± SDs of three independent experiments. ***p < 0.001; Student’s *t*-test. **e** Correlation analysis based on data from public databases. Using the RNA-seq data of liver tissues from the Genotype-Tissue Expression database (*n* = 110), the relative influence between *Cmip* and *Pparγ* expression levels (left panel) and between *Cmip* and *Cd36* expression levels (right panel) was analyzed via Pearson’s correlation analysis. **f** The effect of *Tet2* knockdown on *Cd36* expression. Two sets of *Tet2* siRNA were transiently transfected into AML12 cells, total RNA was extracted, and the mRNA expression of *Cd36* was measured by qRT‒PCR. The values presented are the means ± SDs of three independent experiments. *p < 0.05 and ***p < 0.001; Student’s *t*-test. **g** The effect of the DNMT inhibitor SGI-1027 on *Pparγ* and *Cd36* expression. Cells were treated with SGI-1027 at the indicated concentrations for 24 h. mRNA expression of *Pparγ* (left panel) and *Cd36* (right panel) was measured by qRT‒PCR. The values presented are the means ± SDs of three independent experiments. ***p < 0.001; Student’s *t*-test. **h** The effect of *Cmip* knockdown on fatty acid uptake into AML12 cells. **p < 0.01 and ***p < 0.001; Student’s *t*-test. **i** The effect of Cd36 overexpression under Cmip knockdown conditions. The Cd36 plasmid was transiently transfected following siCmip transfection. After 12 h, OPA was added, and the cells were incubated for an additional 12 h. The rate of fatty acid uptake into AML12 cells was measured. *p < 0.05, **p < 0.01, and ***p < 0.001; Student’s *t*-test.

We confirmed that Cmip was increased in OPA-exposed AML12 cells (Fig. 4a, b). High glucose conditions did not affect Cmip expression in AML12 cells (Supplementary Fig. 10). To explore whether Cmip affected the expression of *Pex11α, Ccnd1, Cebpβ*, and *Pparγ*, the mRNA levels of the genes were measured in AML12 cells with *Cmip* knockdown. As shown in Fig. 4c, the transcriptional activation of *Pparγ* was efficiently decreased in both the absence and presence of OPA with *Cmip* knockdown. Additionally, *Ccnd1* expression was increased in the *Cmip* knockdown groups treated with OPA. However, Cmip did not show a significant effect on the expression levels of *Cebpβ* or *Pex11α* (Supplementary Fig. 11).

We next focused on *Pparγ*, a key regulator of lipogenesis, and observed its transcriptional activation of target genes. Interestingly, we found that *Cd36* was most significantly controlled by *Cmip* (Fig. 4d). In addition, correlation analysis using GTeX human liver transcriptome data showed that the gene expression levels of *Pparγ* and *Cd36* showed significant positive correlations with the gene expression of *Cmip* (*p* < 0.0001, *n* = 110; Fig. 4e). We investigated whether Tet2 is involved in the regulation of *Pparγ* and *Cd36*. Similar to *Cmip* knockdown, the knockdown of *Tet2* dramatically inhibited the expression of *Cd36* (Fig. 4f), and it was reversibly increased following SGI-1027 treatment (Fig. 4g).

As Cd36 impacts lipid uptake, we confirmed the fatty acid uptake into cells. As shown in Fig. 4h, relative to that in the group treated with OPA alone, fatty acid uptake in the *Cmip* siRNA-treated groups following OPA treatment was significantly decreased. *Cd36* overexpression after *Cmip* knockdown showed that fatty acid uptake was regulated in a Cd36-dependent manner upon Cmip knockdown in AML12 cells (Fig. 4i). Taken together, these results indicate that fatty acid-induced *Cmip* facilitates Cd36 expression and fatty acid uptake in a Cd36-dependent manner in vitro.

### Hypomethylation-mediated Cmip expression has a positive correlation with Tet2, Pparγ, and Cd36 expression in *ob/ob* mice

**Fig. 5:**
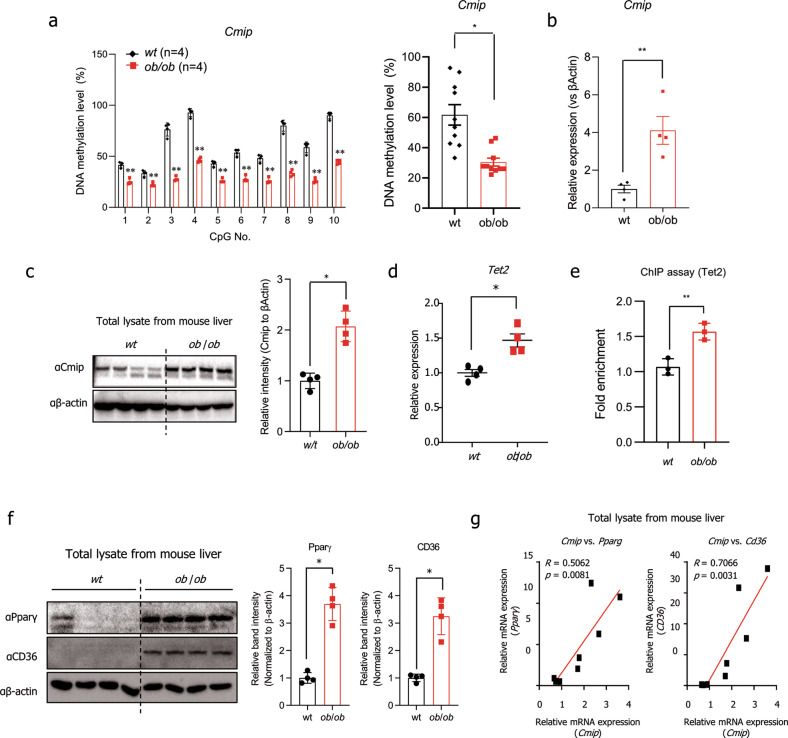
Methylation alteration of *Cmip* controls hepatic lipid accumulation in *ob/ob* mice. **a** The methylation status of ten CpG sites in the *Cmip* intron 1 region in either wildtype (wt) or *ob*/*ob* mouse liver tissues. *Cmip* methylation was measured by bisulfite amplicon sequencing (BSAS) in both wt and *ob*/*ob* mice (*n* = 4/group). The methylation level of each site was calculated (left panel). Total methylation levels between wt and *ob*/*ob* mice were compared (right panel). The values presented are the means ± SEs of four mice per group. **p < 0.01. Student’s *t*-test. **b**, **c** The level of *Cmip* expression in liver tissues from either wt or *ob/ob* mice as measured by qRT‒PCR. The values presented are the means ± SEs of four mice per group. *p < 0.05; Student’s *t*-test (**b**). The protein contents of Cmip were measured by western blotting. Liver lysates were immunoblotted with the indicated antibodies (left panel). The intensities of the protein bands obtained from the western blot assays were quantified using FusionCapt Advance Solo 7 software (right panel) and normalized with respect to the intensity of β-actin. The relative fold intensity was calculated as the sum of the normalized intensities from both β-actin and Cmip. *p < 0.05; Student’s *t*-test (**c**). **d** The expression of *Tet2* in either wt or *ob/ob* mouse liver tissues. *Tet2* mRNA expression was measured by qRT‒PCR. The values presented are the means ± SEs of four mice per group. *p < 0.05; Student’s *t*-test. **e** Tet2 occupancy in the *Cmip* intron 1 region in the livers of *ob/ob* and *wt* mice. The values presented are the means ± SDs of three independent experiments. **p < 0.01; Student’s *t*-test. **f** Pparγ and Cd36 expression in either wt or *ob/ob* mouse liver tissues, detected by western blotting (left panel). The intensities of the protein bands obtained from the western blot assays were quantified with FusionCapt Advance Solo 7 software and normalized with respect to the intensity of β-actin. The relative fold intensity was calculated as the sum of the normalized intensities from β-actin, Pparγ, and Cd36 (right panels). *p < 0.05; Student’s *t*-test. **g** Correlations (R) between Pparγ or Cd36 expression and Cmip expression in ob/ob and wt mouse livers. Pearson’s correlation analysis was performed.

BSAS was conducted to observe the methylation status of the *Cmip* intron 1 region. Interestingly, in liver tissues from *ob/ob* mice, all 10 CpG sites in *Cmip* intron 1 were perfectly hypomethylated (Fig. 5a, left panel), and the total methylation level of the CpG sites decreased by approximately 50% relative to that in wt mice (Fig. 5a, right panel). Supporting the previous results, Cmip expression was significantly higher than that in the wt group (Fig. 5b, c). The mRNA expression of *Tet2* was also higher in *ob/ob* mice than in wt mice (Fig. 5d). In addition, ChIP assays showed that Tet2 was recruited to the CpG region in *Cmip* intron 1 in the livers of *ob/ob* mice (Fig. 5e).

Next, we detected Pparγ and Cd36 expression. As shown in Fig. 5f, compared with wt mice, *ob/ob* mice had higher expression levels of the two proteins in liver tissues (Fig. 5f). We found a strong positive correlation between *Cmip* and *Cd36* expression (*R* = 0.71, *p* = 0.0031) and between *Cmip* and *Pparγ* expression (*R* = 0.51, *p* = 0.0081) (Fig. 5g). These results demonstrated that *Cmip* mRNA expression is induced by CpG hypomethylation of the *Cmip* intron 1 region and has a positive correlation with Tet2, Pparγ, and Cd36 mRNA expression in *ob/ob* mice.

### Knockdown of *Cmip* ameliorates NAFLD pathogenic features in *ob/ob* mice

**Fig. 6:**
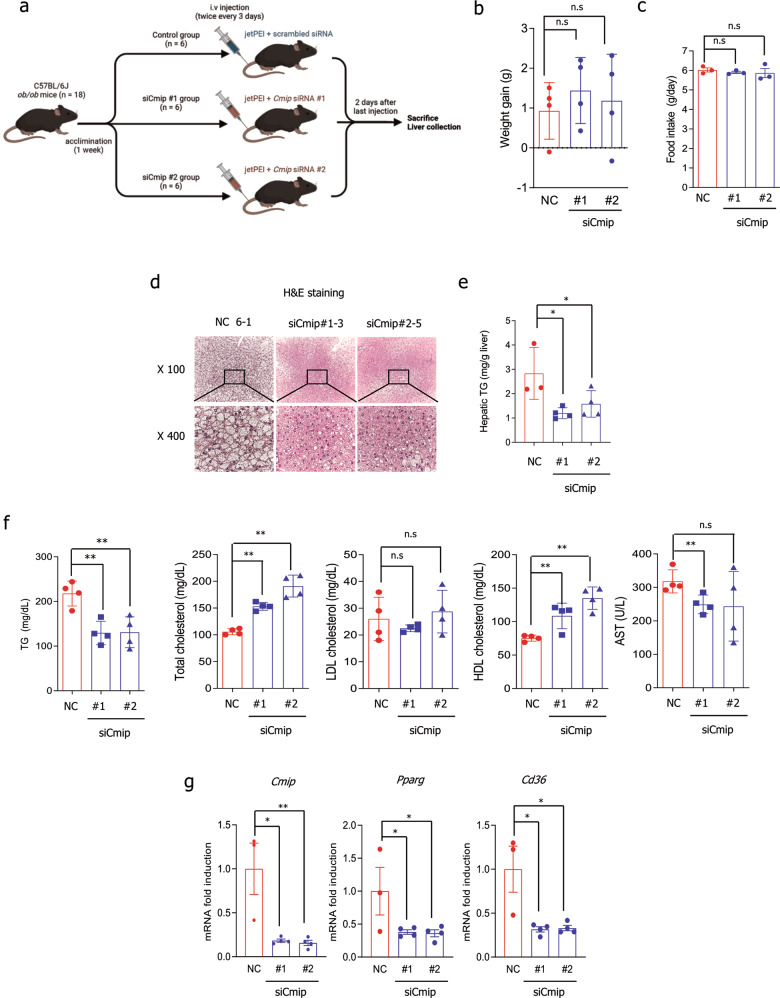
*Cmip* knockdown ameliorates significant nonalcoholic fatty liver disease (NAFLD) pathological features in *ob/ob* mice. **a** Scheme for the in vivo Cmip gene knockdown experiment. Eighteen C57BL6/J ob/ob mice were acclimated for a week and then divided into three groups: the control, siCmip#1, and siCmip#2 groups (six mice per group). The mice were intravenously injected with scrambled siRNA, Cmip siRNA#1, or Cmip siRNA#2 with jetPEI transfection reagents twice every 3 days. Two days after the last injection, the mice were sacrificed, and liver tissue was harvested for analysis. **b**, **c** Measurement of body weight gain and food intake. Negative control (NC; siCont), si*Cmip*#1, or si*Cmip*#2 was intravenously (IV) injected using the jetPEI system twice every 3 days. Body weight gain (**b**) and daily food intake (**c**) for 1 week were calculated, and the values presented are the mean ± SE. *n* = 4/group; n.s. nonsignificant; Student’s *t*-test. **d**–**f** Changes in NAFLD features following *Cmip* knockdown in *ob/ob* mice. Two days after the last IV injection, the mice were sacrificed, the livers were removed, and blood was collected through orbital bleeding. Hematoxylin and eosin (H&E) staining of mouse liver specimens was performed. Representative images at 100× magnification are shown (**d**, upper line), and the center of each image (square block) was observed at 400× magnification (**d**, lower line). Hepatic triglycerides (TGs) and serum levels of TGs total cholesterol, low-density lipoprotein (LDL) cholesterol, high-density lipoprotein (HDL) cholesterol, and aspartate transaminase (AST) were measured (*n* = 6/group) (**f**). The values presented are the mean ± SE. *p < 0.05, **p < 0.01, and n.s. nonsignificant; Student’s *t*-test. **g** mRNA expression of *Cmip*, *Pparg*, and *Cd36* in *Cmip* knockdown *ob/ob* mice as measured by qRT‒PCR (*n* = 4/group). The values presented are the means ± SEs. *p < 0.05, **p < 0.01; Student’s *t*-test.

To further confirm the role of Cmip in NAFLD in vivo, we conducted siRNA-mediated knockdown of *Cmip* in *ob/ob* mice using jetPEI in vivo transfection reagent. Three groups of ob/ob mice (*n* = 6 per group) were intravenously injected with scrambled siRNA or Cmip siRNAs with the reagent twice every 3 days (Fig. 6a). As shown in Fig. 6b, c, neither weight gain nor food intake showed significant differences among mice injected with jetPEI–negative control (NC) or jetPEI–*Cmip* siRNAs. In H&E-stained liver tissue, lipid accumulation was observed to be lower in the jetPEI–*Cmip* siRNA-treated mice than in the jetPEI–NC group, and the lipid droplet size was also decreased in the jetPEI–*Cmip* siRNA groups (Fig. 6d). Injection of jetPEI–*Cmip* siRNAs significantly decreased the hepatic and serum TG levels (Fig. 6e, f, left panel). Total cholesterol was notably enhanced in *Cmip* knockdown mice, with increasing HDL cholesterol but no change in LDL cholesterol (Fig. 6f, middle panels). However, serum aspartate transaminase levels showed a statistically significant decrease only in the jetPEI–si*Cmip*#1-injected mice, and there was no difference in the jetPEI–si*Cmip*#2-injected mice (Fig. 6f, right panel). The qRT‒PCR results showed that *Cmip* was efficiently knocked down through IV injection with the jetPEI system and, supporting the previous results, that the transcriptional activities of *Pparγ* and *Cd36* were diminished following *Cmip* downregulation (Fig. 6g). These results suggest that *Cmip* knockdown in *ob/ob* mice ameliorated *Pparγ* and *Cd36* expression, as well as NAFLD pathogenic features.

### *Gbp2* regulates Pparγ and Cd36 downstream of Cmip

**Fig. 7:**
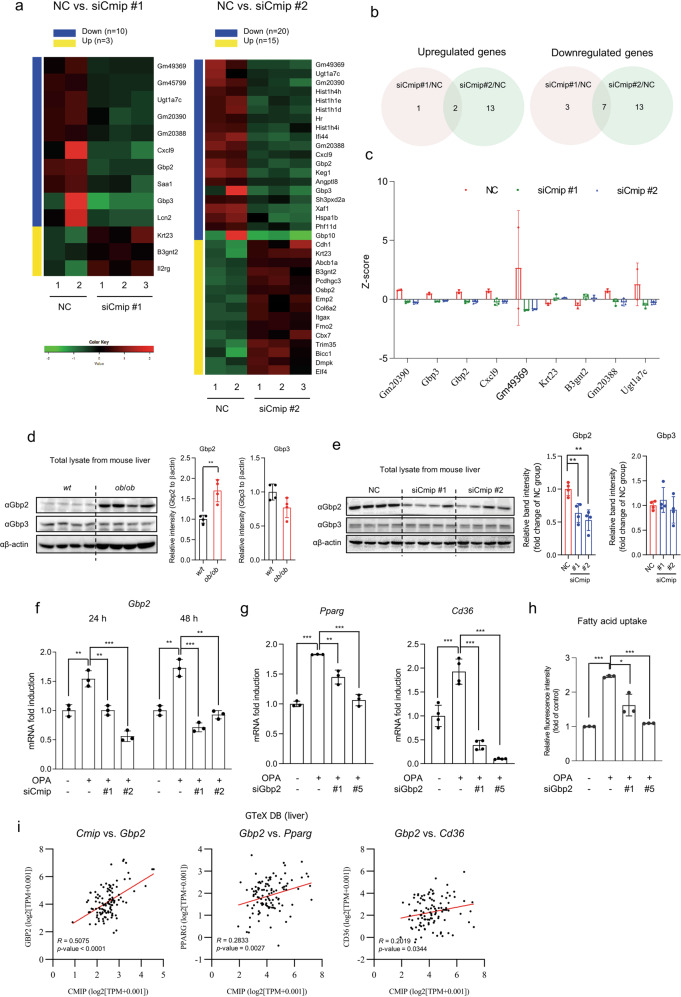
Gbp2 mediates the regulation of the Pparγ*–*Cd36 axis downstream of Cmip. **a** Heatmap visualization of the differentially expressed genes (DEGs) between *Cmip* knockdown and negative control (NC) mice. Two *Cmip* siRNAs (si*Cmip*#1 and si*Cmip*#2) were used for in vivo *Cmip* knockdown. The significant DEGs were selected as follows: |log fold-change|≥1; *p* < 0.05. **b** Venn diagrams representing the overlap of the upregulated DEGs in si*Cmip*#1 vs. NC and si*Cmip*#2 vs. NC (left panel) and that of the downregulated DEGs (right panel). **c** The expression levels of overlapping DEGs (*n* = 9) in the livers of *Cmip* knockdown and NC mice. The expression levels of each gene were expressed as *Z*-scores. **d** Gbp2 and Gbp3 expression in liver tissues in either wildtype (wt) or *ob/ob* mice. The protein levels of Gbp2 and Gbp3 in liver tissues in wt or *ob/ob* mice were detected by western blotting (*n* = 4/group). **p < 0.01; Student’s *t*-test. The intensities of the protein bands obtained from the western blot assays were quantified with FusionCapt Advance Solo 7 software and normalized with respect to the intensity of β-actin. The relative fold intensity was calculated by the sum of normalized intensities from each protein band. *p < 0.05; Student’s *t*-test. **e** Western blots of Gbp2 and Gbp3 expression in *Cmip* knockdown mice (*n* = 4/group). The intensities of the protein bands were quantified with FusionCapt Advance Solo 7 software and normalized with respect to the intensity of β-actin. The relative fold intensity was calculated as the sum of the normalized intensities from each protein band. **p < 0.01; Student’s *t*-test. **f** *Gbp2* mRNA expression in *Cmip* knockdown AML12 cells, measured following si*Cmip* transfection in the presence of oleic acid and palmitic acid (OPA). The values presented are the means ± SDs of three independent experiments. **p < 0.01 and ***p < 0.001; Student’s *t*-test. **g** *Pparγ* and *Cd36* mRNA expression in *Gbp2* knockdown AML12 cells, measured following si*Gbp2* transfection in the presence of OPA. The values presented are the means ± SDs of three independent experiments. **p < 0.01 and ***p < 0.001; Student’s *t*-test. **h** Fatty acid uptake in *Gbp2* knockdown AML12 cells, measured following *Gbp2* siRNA transfection. *p < 0.05, **p < 0.01, and ***p < 0.001; Student’s *t*-test. **i** Correlations of the relative influence between CMIP vs. GBP2, GBP2 vs. PPARγ, and GBP2 vs. CD36 in human livers. Correlations were analyzed using transcriptome data of human liver tissues (*n* = 110) from the Genotype-Tissue Expression (GTeX) database. Correlations are expressed as Pearson correlation coefficients (*R*).

We next sought to find a novel mediator between Cmip and the Pparγ–Cd36 axis by investigating transcriptome changes in the liver tissue of mice administered jetPEI–*Cmip* siRNAs. Three and ten genes were upregulated and downregulated, respectively, in jetPEI–si*Cmip*#1-injected mice (Fig. 7a, left panel). In jetPEI–si*Cmip*#2-injected mice, ten genes were upregulated, and 20 genes were downregulated (Fig. 7b, right panel).

To confirm the reliability of the results, we searched for DEGs common to each group (Fig. 7b). Compared with the jetPEI–NC-treated mouse group, the jetPEI–*Cmip* siRNA-treated mouse groups had two genes (*Krf23* and *B3gnt2*) that were more significantly activated, while seven genes (*Gm20390*, *Gbp3*, *Gbp2*, *Cxcl9*, *Gm49369*, *Gm20388*, and *Ugt1a7c*) were inactivated (Fig. 7c).

Based on recent reports^25,26^, we sought to determine whether Gbp2 or Gbp3 might act as a mediator between Cmip and the Pparγ–Cd36 axis. The results showed that Gbp2 was surprisingly stabilized in liver tissues from *ob/ob* mice (Fig. 7d). However, there was no difference in Gbp3 expression. In addition, *Gbp2* and *Pparg* expression was higher in HFD-fed mice than in ND-fed mice in publicly available DBs (GSE95428 and GDS6248) (Supplementary Fig. 12). Next, we observed Gbp2 and Gbp3 expression in liver tissues of *ob/ob* mice administered either jetPEI–NC or jetPEI–*Cmip* siRNAs. Interestingly, Gbp2 expression was significantly decreased in the livers of mice injected with jetPEI–*Cmip* siRNAs (Fig. 7e); however, Gbp3 showed no change. A decrease in *Gbp2* following *Cmip* knockdown was also verified in OPA-exposed AML12 cells (Fig. 7f). Furthermore, *Pparγ* and *Cd36* expression was downregulated in si*Gbp2*-transfected AML12 cells relative to that in OPA-treated cells (Fig. 7g), consequently decreasing fatty acid uptake; OPA-enhanced fatty acid uptake also decreased following *Gbp2* knockdown (Fig. 7h).

We analyzed the relative influence between the expression of *Gbp2* and that of *Cmip*, *Pparγ*, or *Cd36* through Pearson correlation analysis using GTeX transcriptome data. As shown in Fig. 7i, the expression of *Gbp2* was positively correlated with that of *Cmip*, and the correlations with *Pparγ* and *Cd36* expression levels were positive but weaker. These data suggest that Gbp2 but not Gbp3 could be a potential Cmip downstream mediator that regulates the expression of *Pparγ* and *Cd36* in *ob/ob* mice.

### Cmip, Gbp2, Pparγ, and Cd36 are overexpressed in liver tissues of patients with NAFLD

**Fig. 8:**
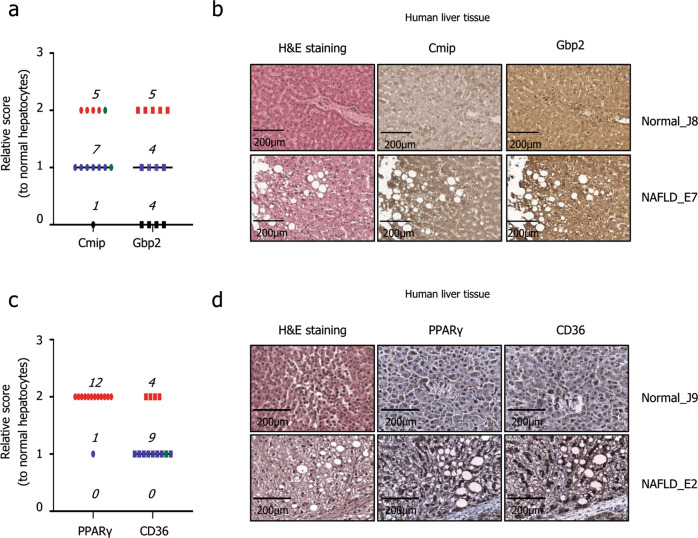
The expression of Cmip, Gbp2, Pparγ, and Cd36 in human hepatocytes with nonalcoholic fatty liver disease (NAFLD). **a** Cmip and Gbp2 expression levels in the livers of patients with NAFLD compared with those in normal livers. Score 0, black circle; Score 1, blue circle; Score 3, red circle; focal score, green circle. **b** Representative images of Cmip and Gbp2 stained with hematoxylin and eosin. Scale bar = 200 µm. **c** Pparγ and Cd36 expression levels in the livers of patients with NAFLD compared with those in normal livers. Score 0, black circle; Score 1, blue circle; Score 3, red circle; focal score, green circle. **d** Representative images of Cmip and Gbp2 stained with hematoxylin and eosin. Scale bar = 200 µm.

Finally, to demonstrate the potential of *Cmip* as a novel target for NAFLD therapies, we explored Cmip and Gbp2 expression in human liver tissues. Cmip was highly expressed in 12 of the 13 NAFLD tissues. One of two samples with focal scores (green) comparable to those in normal tissues also had a high expression level. Gbp2 was observed to be highly expressed in nine NAFLD tissues, and in four tissues, it presented a similar expression level to that in normal tissues (Fig. 8a, b and Supplementary Tables 9, 10). In liver tissues with NAFLD, Cmip expression was 92.3% higher and Gbp2 expression was 76.9% higher than the expression in normal tissues. The high expression of Pparγ and Cd36 in liver tissues of patients with NAFLD is already well known. To ensure the reliability of the results shown above, we observed the expression of Pparγ and Cd36 in the same tissue. Both proteins showed a high expression pattern in all 12 NAFLD liver tissues compared to normal tissues (Fig. 8c, d and Supplementary Tables 9, 10).

## Discussion

NAFLD, defined as a pathologic accumulation of TGs within hepatocytes not caused by significant alcohol consumption, is the most common cause of chronic liver diseases worldwide^2,27^. The mechanisms underlying the development and progression of NAFLD have not been entirely elucidated. However, recent studies have described a new model in which multiple parallel hits are responsible for the pathogenesis of NAFLD^28,29^. More recently, the effects of nutrition on the development and progression of NAFLD through epigenetic regulation have been highlighted^30,31^. In particular, aberrant methylation of either genomic or mitochondrial DNA causes abnormal gene expression in NAFLD^32,33^. In this study, we identified supporting evidence that diet-induced epigenetic changes have a close association with NAFLD. To our knowledge, this is the first study to demonstrate the epigenetic regulatory mechanism of *Cmip* and its involvement in NAFLD.

Previous studies have reported an association between *Cmip* and metabolic diseases. Cao et al., in 2018, reported that *Cmip* may exert independent pleiotropic effects on T2DM and obesity-related phenotypes in females^18^. In 2017, Sayols-Baixeras et al. demonstrated that *Cmip* is involved in the regulation of HDL cholesterol efflux capacity^34^. The latter is the only study reported to date that suggests the possibility of controlling HDL cholesterol efflux through epigenetic modification of *Cmip*. Although two methylation sites related to cholesterol efflux capacity in peripheral blood cells were found in *Cmip*, a previous study did not elucidate the functional mechanisms of *Cmip* methylation that underlie the regulation of HDL cholesterol.

Alteration of the methylation status of the *Cmip* intron 1 region directly affects the expression of genes. In both in vitro and in vivo control models, hypermethylated *Cmip* shows low gene expression, whereas, in NAFLD models, hypomethylated *Cmip* is highly expressed. DNMT1 has a high affinity for hemimethylated DNA^35^, causing repression of associated genes^1^. The results of our siRNA experiments and ChIP assays support these findings. Although the knockdown of Dnmt3b also increased Cmip expression, similar to that of Dnmt1, Dnmt3b did not occupy the CpG region in Cmip intron 1. Dnmt3b is associated with *de novo* DNA methylation, which is a process by which methyl groups are added to unmethylated DNA at specific CpG sites^36^. Our data clearly show that CpG sites in the region are already methylated under normal conditions, indicating that it is impossible for Dnmt3b to access the methylated region. H3K27me3 orchestrates the epigenetic silencing of genes through complex formation with DNMT1^37^. Additionally, H3K27me3 regions are frequent targets for DNA methylation^38^. Our results also strongly confirmed those of previous reports by demonstrating a decrease in peripheral H3K27me3 with Dnmt1 dissociation from the *Cmip* intron 1 region.

Generally, methylation of CpG islands in the DNA promoter is well known to directly prevent transcription factor binding and lead to changes in chromatin structure that indirectly restrict access of transcription factors to the gene promoter, consequently inhibiting gene expression^39,40^. However, according to our results, *Cmip* methylation and the various biological events associated with it occur in *Cmip* intron 1 rather than in its promoter region. In fact, the methylation status of the intron is also involved in controlling the transcription of some genes. For example, intron 1 of triggering receptor expressed on myeloid cells 2 shows a lower methylation rate in patients with schizophrenia than in patients without schizophrenia, and it has a significant negative correlation with triggering receptor expressed on myeloid cells 2 mRNA expression^41^. Increases in *SNCA* (alpha-synuclein)^42,43^, *NR5A1* (steroidogenic factor 1)^44^, and *PXMP4* (peroxisomal membrane protein 4)^45^ mRNA expression are also due in part to DNA methylation of intron 1.

The high contents of C + G, CG dinucleotides, and unmethylated CpG islands could provide potential binding sites for some important transcription factors^46^. For example, the binding of Ctcf^47^ or Sp1^48^ to intron 1 is dependent on DNA methylation, suggesting that the methylation status surrounding the spliced exons could affect the inclusion level of these exons. Previous studies have verified the Ctcf-dependent performance of Tet2^49,50^. In particular, the binding of Ctcf, a multifunctional transcription factor, reduces the local methylation level, presumably by the recruitment of Tet2, which can demethylate surrounding CpG sites^40^. Furthermore, the association between Ctcf and Tet was recently experimentally validated^23^.

Based on previous studies, we investigated the involvement of Tet2 and Ctcf in *Cmip* hydroxymethylation and activation. Our results suggest that Tet2 might be involved in *Cmip* hydroxymethylation through Ctcf mediation, considering the strong positive correlation between Cmip and Tet2. With the involvement of Tet2 and Ctcf, peripheral H3K4me3, a gene activation marker^51^, was also shown to be increased in the *Cmip* intron 1 region. The results described thus far show that *Cmip* expression is reversibly regulated by methylation changes mediating various factors, such as Dnmt1, Tet2, and Ctcf.

As stated earlier, some previous studies have indicated a relationship between *Cmip* and metabolic diseases, but no study directly demonstrating the involvement of *Cmip* in NAFLD has been reported yet. In GO enrichment analysis based on two open datasets, we observed the effects of *Cmip* on four genes, namely, *Pparγ*, *Cebpb*, *Pexl1a*, and *Ccnd1*, with the highest expression levels in mice fed an HFD; however, our data clearly showed that knockdown of *Cmip* interrupted *Pparγ* expression, suggesting that *Cmip* is responsible for the development of NAFLD. *Pparγ* is considered to be a master regulator due to its widespread influence on the control of lipid uptake, transport, storage, and disposal in the process of lipid metabolism^52^. *Ccnd1* has been well established as a negative regulator of adipogenesis^53^. Furthermore, in the presence of glucose, the expression of *Ccnd1*, which shows the opposite trend to that of *Pparγ*, represses the transcription of lipogenesis-related genes, particularly *Fas* and *Acc*, in hepatocytes^54^. Additionally, previous reports have demonstrated the repressive effect of *Ccnd1* on lipogenesis through suppression of either *Pparγ*^53,54^ or hepatocyte nuclear factor 4α^55^. These reports strongly support our hypothesis that *Cmip* contributes to the development and progression of NAFLD.

Cd36, a hepatic fatty acid transporter, is reported to be a hepatocyte-specific target of Pparγ that promotes steatosis^54^. The results of our analysis of cellular fatty acid uptake reveal that *Cmip* is a key factor for regulating hepatic lipid accumulation through the Pparγ–Cd36 axis, which is a lipid transport signaling pathway. Furthermore, we IV injected *Cmip* siRNAs into a NAFLD *ob/ob* mouse model. Previously, we mentioned a study that suggested the possibility of HDL cholesterol regulation by Cmip methylation^34^. Although the study did not detail the underlying mechanism, we cautiously speculate that the mechanism is also regulated through the Pparγ–Cd36 signaling pathway.

Moreover, this study used transcriptome analysis to identify Gbp2 as a novel factor that mediates Cmip and the Pparγ–Cd36 axis. A member of the family of IFN-induced GTPases, Gbp2 forms inflammasomes and activates immune signaling downstream of IFN receptors^56^. However, its role in fatty degeneration was previously unknown. Although a mouse liver transcriptome analysis study showed that Gbp2 is one of the activated genes in HFD-fed mice^26^, the study did not meaningfully address the role of Gbp2. Our study clearly demonstrates that the high expression of Gbp2 in the liver tissues of *ob/ob* mice is positively correlated with that of Cmip.

In obesity, proinflammatory T-helper 1 cells and classically activated macrophages (M1) are activated and produce various inflammatory cytokines, such as IFN-γ, tumor necrosis factor-α, and interleukin-12^57,58^. IFN-γ and granulocyte–macrophage colony-stimulating factor induce M1 macrophages^57,59^. Based on previous studies reporting the role of IFN-γ in obesity and the correlation between Gbps and IFN-γ, the potential for Gbp2 to be involved in the regulatory mechanisms in NAFLD is plausible, although the detailed underlying mechanisms remain unknown. Given the well-known role of Gbp2, it may be possible to prevent the transition from NAFLD to NASH by inhibiting Gbp2 or by downregulating Cmip. Gbp2 targeting may also be considered for therapeutic application for NASH patients.

Based on these findings, we compared Cmip and Gbp2 expression between normal and NASH livers from mice fed either an ND or a choline-deficient l-amino acid-defined HFD. Interestingly, in NASH livers, the mRNA and protein expression levels of Cmip and Gbp2 were significantly stabilized (Supplementary Fig. 13). However, to support the aforementioned hypotheses, a further in-depth study should be conducted. Finally, by demonstrating overexpression of Cmip and Gbp2 in the hepatocytes of patients with NAFLD, we suggest a reasonable possibility that both proteins could be effective targets for NAFLD prevention and treatment.

Our study has certain limitations. First, our study did not exhaustively investigate the regulatory mechanism of Tet2 and Ctcf at the *Cmip* intron 1 region. Second, we attempted to validate our hypothesis using various methods, but in vivo validation via the conditional knockdown system was not performed due to the lack of a model. Third, we also found for the first time that Gbp2 is regulated by Cmip in NAFLD models and subsequently activates the Pparγ–Cd36 axis, but the underlying mechanisms were not fully observed. Further relevant in-depth studies to overcome these limitations should be conducted.

**Fig. 9:**
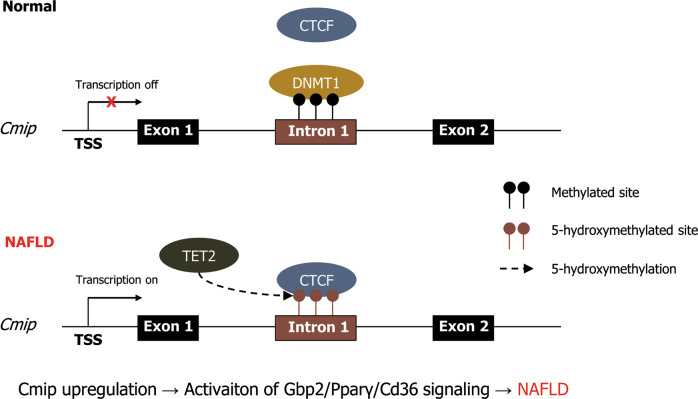
Schematic representation of the core contents in this study. The potential mode of action of Cmip in nonalcoholic fatty liver disease (NAFLD) development. The *C*mip intron 1 region is hypermethylated by Dnmt1 in normal liver tissues; however, following lipid accumulation in the liver, Tet2 is recruited with Ctcf and alters the methylation status in that region of *Cmip*, sequentially increasing the expression of both its mRNA and protein levels. Finally, increased Cmip activates the signaling pathway involved in the Pparγ–Cd36 axis via Gbp2, consequently accelerating hepatic lipid uptake into cells. Thus, Cmip represents a promising novel target for the prevention and treatment of NAFLD. 5mc 5-methylcytosine.

In summary, we have elucidated for the first time the regulatory mechanism of *Cmip* expression and its effect on the development and progression of NAFLD (Fig. 9). We conclude the following: (i) *Cmip* expression is regulated by altering its methylation, and Dnmt and Tet2 are the major enzymes involved; (ii) hypomethylation enhances *Cmip* expression and facilitates the development and progression of NAFLD by activating the Pparγ–CD36 signaling pathway; and (iii) Gbp2, a newly identified factor mediating Cmip and the Pparγ–Cd36 axis, is also responsible for NAFLD, indicating that Cmip and Gbp2 are new preventive and therapeutic targets for NAFLD.

## Supplementary information

Supplemental Materials
