# Supplementary material for: Dnmt1/Tet2-mediated changes in Cmip methylation regulate the development of nonalcoholic fatty liver disease by controlling the Gbp2-Pparγ-CD36 axis
Source: Exp Mol Med. 2023 Jan 6;55(1):143–57. doi: 10.1038/s12276-022-00919-5 (PMC9898513; doi:10.1038/s12276-022-00919-5)
Supplement: Supplementary file 1 — Supplemental Materials [file 12276_2022_919_MOESM1_ESM.pdf]

Supplemental Materials

Materials and Methods

Animal experiments

Forty male C57BL/6N mice (7 weeks old, 22–25 g) were divided into four groups and housed in a temperature- and humidity-controlled room with a 12-h light/12-h dark cycle. After acclimation for one week, the groups were fed either an HFHS diet (D12079B, Research Diets, New Brunswick, NJ, USA) or a normal diet (ND) (98052602, Research Diets) for 12 weeks. At the end of the experiment, the mice were sacrificed, and livers were collected and stored at –80 ℃ for further assessment. Animal experiments using high-fat high-sucrose (HFHS) diet-fed mice were conducted according to the Guide for the Korea Food Research Institutional Animal Care and Use of Committee (KFRI-M-15012). The animal experiments using C57BL/6J-*wt* (wt) and C57BL/6J-*ob*/*ob* (*ob*/*ob*) mice were performed in accordance with the National Institute of Health Guide for the Care and Use of Laboratory Animals and were approved by the Ulsan University Guide for the Animals Care and Use Committee (2019-02-361). For the experimets of comparision between *wt* and *ob*/*ob*, the mice were sacrificed after one week of acclimation, and used for experiments. For siRNA inoculation, 18 male *ob*/*ob* mice were divided into three groups (*n* = 6 each) and housed in a temperature- and humidity-controlled room with a 12-h light/12-h dark cycle. After a one-week acclimation period, 50 μg siRNA (siCont, si*Cmip*#1, or si*Cmip*#2) was intravenously (IV) injected with jetPEI (Polyplus-transfection, New York, NY, USA) twice every 3 days according to the manufacturer's protocol. Two days after the last injection, mice were sacrified by cervical dislocation under anesthesia (100 mg/kg katamine + 5 mg/kg xylazine), and livers were used for the indicated experiments.

Supplementary Tables

Supplementary Table 1. List of antibodies used for western blotting and ChIP assays

	Vendor<br>Cat no.	Local	Country	Titer	Usage
αCMIP	Proteintech<br>12851-1-AP	Danvers, MA	USA	1:1000	Western blotting
αDNMT1	Cell signaling<br>5032S	Danvers, MA	USA	1:1000	
αTET2	Abcam<br>Ab94580	Cambridge, MA	USA	1:1000	
αDNMT3a	Cell signaling<br>2160S	Danvers, MA	USA	1:1000	
αDNMT3b	Abcam<br>Ab16049	Cambridge, MA	USA	1:1000	
αPPARγ	Bethyl Laboratories<br>A304-460A	Montgomery, Texas	USA	1:1000	
αCD36	Novus Biological<br>NB400-144	Littleton, CO	USA	1:1000	
αβActin	Santa Cruz Biotechnology<br>sc-47778	Dallas, Texas	USA	1:5000	
	Cell signaling<br>4967L	Danvers, MA	USA	1:3000	
αDNMT1	Active Motif<br>39204	Carlsbad, CA	USA	1:100	ChIP
αDNMT3b	Cell signaling<br>48488S	Danvers, MA	USA	1:50	
αH3K27me3	Cell signaling<br>9733S	Danvers, MA	USA	1:50	
αTET2	Cell signaling<br>92529S	Danvers, MA	USA	1:50	
αH3K4me3	Cell signaling<br>9751S	Danvers, MA	USA	1:50	
α5-hmC	Active motif<br>39770	Carlsbad, CA	USA	1:50	
αCTCF	Cell signaling<br>3418S	Danvers, MA	USA	1:50	

Supplementary Table 2. Primer sequences used for qRT-PCR and ChIP assay

Gene	Primer sequence		Usage
	Forward	Reverse	
Cmip	AAC GGG ATG AGG TAC AAG CTG	GGA TGC TAA GCT GTT GTC GG	qRT-PCR
Dnmt1	ATC CTG TGA AAG AGA ACC CTG T	CCG ATG CGA TAG GGC TCT G	
Pparγ	GCG GGC TGA GAA GTC ACG TT	CCA TCA CGG AGA GGT CCA CA	
CD36	TGC ACC ACA TAT CTA CCA AA	TTG TAA CCC CAC AAG AGT TC	
Cebpβ	AAG ATG CGC AAC CTG GAG A	TTG AAC AAG TTC CGC AGG G	
Pex11a	GAC GCC TTC ATC CGA GTC G	CGG CCT CTT TGT CAG CTT TAG A	
Ccnd1	GCG TAC CCT GAC ACC AAT CTC	CTC CTC TTC GCA CTT CTG CTC	
Tet2	AGA GAA GAC AAT CGA GAA GTC GG	CCT TCC GTA CTC CCA AAC TCA T	
Gbp2	CTG CAC TAT GTG ACG GAG CTA	GAG TCC ACA CAA AGG TTG GAA A	
Actb	CGC CAC CAG TTC GCC ATG GA	TAC AGC CCG GGG AGC ATC GT	
Cmip (intron 1)	ACT GCT GCC CTC ATT TCA CAG	TGG TGG AGC AGT TTC AGA TGG	ChIP

Supplementary Table 3. List of siRNAs and their sequences used for this study

Target gene	siRNA name	Duplex sequence (5' -> 3')	
CMIP	Cmip #1	Sense	GCUUCAACCCAUUCCAUUCUU
		Antisense	GAAUGGAAUGGGUUGAAGCUU
	Cmip #2	Sense	CAAAGGAACUGAAGUACGUUU
		Antisense	ACGUACUUCAGUUCCUUUGUU
DNMT1	Dnmt1 #1	Sense	GGAAAGAGAUGGCUUAACAUU
		Antisense	UGUUAAGCCAUCUCUUUCCUU
	Dnmt1 #3	Sense	UCAUUGAGGCGGAAAUCAAUU
		Antisense	UUGAUUUCCGCCUCAAUGAUU
DNMT3a	Dnmt3a #1	Sense	GCAGAACAAGCAGAUGAUUUU
		Antisense	AAUCAUCUGCUUGUUCUGCUU
	Dnmt3a #2	Sense	GCCAAGAAACCCAGAAAGAUU
		Antisense	UCUUUCUGGGUUUCUUGGCUU
DNMT3b	Dnmt3b #4	Sense	GCAAUGAUCUCUCUAACGUUU
		Antisense	ACGUUAGAGAGAUCAUUGCUU
	Dnmt3b #5	Sense	GGAUGUUCGAGAAUGUUGUUU
		Antisense	ACAACAUUCUCGAACAUCCUU
TET2	Tet2 #4	Sense	CCAAAGAGCAUCAUUGAGAUU
		Antisense	UCUCAAUGAUGCUCUUUGGUU
	Tet2 #5	Sense	GUGAAAGUGCCAACAGAUAUU
		Antisense	UAUCUGUUGGCACUUUCACUU
Gbp2	Gbp2 #1	Sense	CUGAUCUUAUCAAGUCAAAUU
		Antisense	UUUGACUUGAUAAGAUCAGUU
	Gbp2 #5	Sense	GCAUCUUCAGGCUUUGAAAUU
		Antisense	UUUCAAAGCCUGAAGAUGCUU
	Negative control	Sense	CCUCGUGCCGUUCCAUCAGGUAGUU
		Antisense	CUACCUGAUGGAACGGCACGAGGUU

Supplementary Table 4. List of publicly-available transcriptome datasets of high-fat diet-induced NAFLD animal experiments.

Organism	Collection	Description	Database
mouse	GDS	51 Illumina MouseWG-6 v2.0 expression beadchip arrays. Diet-induced obesity model: liver. Analysis of livers of C57BL/6J mice fed a high fat diet for up to 24 weeks. Significant body weight gain was observed after 4 weeks. Results provide insight into the effect of high fat diets on metabolism in the liver.	ID=gdsGDS6248
	GREIN	23 mouse RNA-Seq samples. NAFLD in IL-4 and IL-10/4 KO Mice	ID=greinGSE95428

Supplementary Table 5. CpGs in the annotated DMR region

Gene		Gfi1	Snx20	Alox5ap	Mir126b	Dio3os	Bin2	Ripor3	Stat3	Pex26	Cmip	Tle2	Ildr1	Lzts3	Man1c1
Chromosome		5	8	5	2	12	15	2	11	6	8	10	16	2	4
Cpg No.	1	107,721,503	88,627,471	149,287,629	26,591,340	110,220,412	100,669,454	167,980,335	100,918,962	121,187,360	117,325,154	81,580,338	36,709,547	130,635,993	134,720,254
	2	107,721,520	88,627,477	149,287,632	26,591,343	110,220,423	100,669,467	167,980,339	100,918,986	121,187,376	117,325,176	81,580,382	36,709,552	130,636,000	134,720,274
	3	107,721,524	88,627,479	149,287,665	26,591,354	110,220,427	100,669,472	167,980,351	100,919,008	121,187,383	117,325,179	81,580,438	36,709,557	130,636,011	134,720,280
	4	107,721,528	88,627,481	149,287,671	26,591,382	110,220,442	100,669,476	167,980,354	100,919,032	121,187,399	117,325,186	81,580,445	36,709,562	130,636,029	134,720,286
	5	107,721,537	88,627,494	149,287,694	26,591,384	110,220,444	100,669,499	167,980,368	-	121,187,404	117,325,224	81,580,452	36,709,565	130,636,051	134,720,306
	6	107,721,541	88,627,498	149,287,703	26,591,403	110,220,471	100,669,505	167,980,380	-	121,187,418	117,325,243	81,580,503	36,709,582	130,636,060	134,720,319
	7	107,721,544	88,627,500	149,287,706	26,591,408	110,220,478	100,669,511	167,980,403	-	121,187,434	117,325,249	81,580,513	36,709,601	130,636,077	134,720,341
	8	107,721,550	88,627,502	149,287,715	26,591,422	110,220,492	100,669,555	167,980,467	-	121,187,441	117,325,271	81,580,522	36,709,607	130,636,080	134,720,345
	9	107,721,552	88,627,504	149,287,755	26,591,424	110,220,514	100,669,575	167,980,484	-	121,187,451	117,325,289	81,580,536	36,709,628	130,636,116	134,720,347
	10	107,721,558	88,627,506	149,287,776	26,591,439	110,220,554	100,669,591	167,980,491	-	121,187,461	117,325,314	81,580,538	36,709,639	130,636,139	-
	11	107,721,561	88,627,515	149,287,786	26,591,453	-	100,669,625	167,980,495	-	121,187,471	-	81,580,546	36,709,679	130,636,148	-
	12	107,721,577	88,627,518	149,287,791	26,591,472	-	100,669,631	167,980,524	-	121,187,494	-	81,580,561	36,709,682	130,636,189	-
	13	107,721,582	88,627,520	149,287,794	26,591,483	-	100,669,658	-	-	121,187,507	-	-	36,709,686	130,636,198	-
	14	107,721,591	88,627,530	149,287,796	26,591,493	-	-	-	-	121,187,519	-	-	-	130,636,218	-
	15	107,721,597	88,627,539	149,287,848	-	-	-	-	-	121,187,531	-	-	-	130,636,235	-
	16	107,721,600	88,627,541	149,287,865	-	-	-	-	-	-	-	-	-	130,636,257	-
	17	107,721,606	88,627,548	149,287,882	-	-	-	-	-	-	-	-	-	-	-
	18	107,721,625	88,627,553	149,287,917	-	-	-	-	-	-	-	-	-	-	-
	19	107,721,630	88,627,555	149,287,923	-	-	-	-	-	-	-	-	-	-	-

	20	107,721,636	88,627,566	149,287,926	-	-	-	-	-	-	-	-	-	-	-
	21	107,721,640	88,627,575	-	-	-	-	-	-	-	-	-	-	-	-
	22	107,721,651	88,627,577	-	-	-	-	-	-	-	-	-	-	-	-
	23	107,721,657	88,627,579	-	-	-	-	-	-	-	-	-	-	-	-
	24	107,721,662	88,627,583	-	-	-	-	-	-	-	-	-	-	-	-
	25	107,721,664	88,627,586	-	-	-	-	-	-	-	-	-	-	-	-
	26	107,721,670	88,627,590	-	-	-	-	-	-	-	-	-	-	-	-
	27	107,721,674	88,627,593	-	-	-	-	-	-	-	-	-	-	-	-
	28	107,721,679	88,627,595	-	-	-	-	-	-	-	-	-	-	-	-
	29	107,721,685	88,627,599	-	-	-	-	-	-	-	-	-	-	-	-
	30	107,721,690	88,627,602	-	-	-	-	-	-	-	-	-	-	-	-
	31	107,721,692	88,627,613	-	-	-	-	-	-	-	-	-	-	-	-
	32	107,721,696	88,627,623	-	-	-	-	-	-	-	-	-	-	-	-
	33	107,721,715	88,627,625	-	-	-	-	-	-	-	-	-	-	-	-
	34	107,721,717	88,627,634	-	-	-	-	-	-	-	-	-	-	-	-
	35	107,721,722	88,627,637	-	-	-	-	-	-	-	-	-	-	-	-
	36	107,721,730	88,627,640	-	-	-	-	-	-	-	-	-	-	-	-
	37	107,721,732	88,627,644	-	-	-	-	-	-	-	-	-	-	-	-
	38	107,721,743	88,627,656	-	-	-	-	-	-	-	-	-	-	-	-
	39	107,721,764	88,627,660	-	-	-	-	-	-	-	-	-	-	-	-
	40	107,721,786	88,627,671	-	-	-	-	-	-	-	-	-	-	-	-

	41	107,721,814	88,627,679	-	-	-	-	-	-	-	-	-	-	-	-
	42	107,721,831	88,627,685	-	-	-	-	-	-	-	-	-	-	-	-
	43	107,721,842	88,627,687	-	-	-	-	-	-	-	-	-	-	-	-
	44	107,721,859	88,627,693	-	-	-	-	-	-	-	-	-	-	-	-
	45	107,721,862	88,627,698	-	-	-	-	-	-	-	-	-	-	-	-
	46	-	88,627,711	-	-	-	-	-	-	-	-	-	-	-	-
	47	-	88,627,716	-	-	-	-	-	-	-	-	-	-	-	-
	48	-	88,627,721	-	-	-	-	-	-	-	-	-	-	-	-

Supplementary Table 6. The correlation analysis between the expression of the genes involved in demethylation processes and Cmip expression by using publicly-available datasets, TCGA and GTeX data.

Type	Function	Matched Gene	TCGA (n=373)			GTeX (n=110)	
			Cytoband	Pearson's Correlation	p-Value	Pearson's Correlation	p-Value
**Methylcytosine dioxygenase**	**Oxidation**	**TET2**	**4q24**	**0.52**	**3.94E-27**	**0.55**	**4.00E-10**
Methylcytosine dioxygenase	Oxidation	TET3	2p13.1	0.44	2.81E-19	0.4	1.20E-05
DNA glycosylase	Base excision repair (BER)	SMUG1	12q13.13	-0.37	9.21E-14	0.23	0.014
Cytidine deaminase	Deamination	APOBEC3C	22q13.1	0.34	8.29E-12	0.61	1.10E-12
DNA glycosylase	Base excision repair (BER)	TDG	12q23.3	0.18	6.01E-04	0.44	1.40E-06
Cytidine deaminase	Deamination	APOBEC4	1q25.3	0.12	0.0201	0.12	0.21
Methylcytosine dioxygenase	Oxidation	TET1	10q21.3	0.11	0.0366	0.42	6.10E-06
Cytidine deaminase	Deamination	APOBEC1	12p13.31	0.11	0.0423	0.0098	0.92
DNA glycosylase	Base excision repair (BER)	MBD4	3q21.3	0.1	0.0572	0.56	1.40E-10
Cytidine deaminase	Deamination	APOBEC2	6p21.1	0.08	0.148	0.049	0.61
Cytidine deaminase	Deamination	APOBEC3F	22q13.1	-0.07	0.165	0.58	4.90E-11
Cytidine deaminase	Deamination	APOBEC3D	22q13.1	-0.02	0.676	0.5	2.40E-08
Cytidine deaminase	Deamination	APOBEC3A	22q13.1	0.02	0.691	0.33	0.00051
Cytidine deaminase	Deamination	APOBEC3B	22q13.1	-0.01	0.886	0.44	2.00E-06
Cytidine deaminase	Deamination	APOBEC3G	22q13.1	0	0.935	0.62	4.00E-13
Cytidine deaminase	Deamination	APOBEC3H	22q13.1	0	0.957	0.62	4.20E-13

Supplementary Table 7. GO enrichment analysis

Expression	Term	p-value	Genes
Up	fat cell differentiation	0.0014	PEX11A, ADRB2, CCND1, CEBPB, PPARG
	sphingolipid metabolic process	0.0015	PSAP, HEXA, HEXB, SMPD3, CLN6
	brown fat cell differentiation	0.0016	PEX11A, ADRB2, CEBPB, PPARG
	membrane lipid metabolic process	0.0017	PSAP, HEXA, HEXB, SMPD3, CLN6
	ganglioside metabolic process	0.0022	HEXA, HEXB, CLN6
	hexose metabolic process	0.0024	ALDOA, GALK1, PCX, GNPDA1, PFKL, GAPDH, PMM1
	DNA replication initiation	0.0028	MCM2, MCM4, MCM6
	DNA unwinding during replication	0.0033	MCM2, MCM4, MCM6
	DNA duplex unwinding	0.004	MCM2, MCM4, MCM6
	carbohydrate catabolic process	0.0041	ALDOA, GNPDA1, PFKL, HEXB, GAPDH
Down	sulfur amino acid metabolic process	0.0002	CTH, AHCY, GNMT, CBS
	vitamin metabolic process	0.004	ASPDH, RDH11, KYNU, ADH7
	secondary metabolic process	0.004	ASPDH, RDH11, KYNU, ADH7
	organic acid catabolic process	0.0063	HACL1, KYNU, AHCY, FTCD
	carboxylic acid catabolic process	0.0063	HACL1, KYNU, AHCY, FTCD
	cysteine biosynthetic process	0.0093	CTH, CBS
	sulfur metabolic process	0.0094	CTH, AHCY, GNMT, CBS
	monohydric alcohol metabolic process	0.0184	ADH4, ADH7
	ethanol metabolic process	0.0184	ADH4, ADH7
	organophosphate catabolic process	0.0184	ASPDH, NUDT7

* 86 upregulated and 144 downregulated genes were subjected to GO enrichment analysis ($p < 0.01$ cutoff)

Supplementary Table 8. The list of genes for GO enrichment analysis (related to Supplementary Fig.)

	Gene symbol	Entrez ID	Gene name
Up	PLVAP	84094	plasmalemma vesicle associated protein
	APOL9A	223672	apolipoprotein L 9a
	SPARC	20692	secreted acidic cysteine rich glycoprotein
	CLSTN3	232370	calsyntenin 3
	RNF19B	75234	ring finger protein 19B
	CMIP	74440	c-Maf inducing protein
	RAB43	69834	RAB43, member RAS oncogene family
	CCND1	12443	cyclin D1
	RASSF4	213391	Ras association
	CHCHD6	66098	coiled-coil-helix-coiled-coil-helix domain containing 6
	ENPP2	18606	ectonucleotide pyrophosphatase/phosphodiesterase 2
	MB21D2	239796	Mab-21 domain containing 2
	ATP6V1E1	11973	ATPase, H+ transporting, lysosomal V1 subunit E1
	HMGN2	15331	high mobility group nucleosomal binding domain 2
	ACAD10	71985	acyl-Coenzyme A dehydrogenase family, member 10
	TGM2	21817	transglutaminase 2, C polypeptide
	1810058I24RIK	67705	RIKEN cDNA 1810058I24 gene
	TGTP1	21822	T cell specific GTPase 1
	WNT5B	22419	wingless-type MMTV integration site family, member 5B
	APLP2	11804	amyloid beta
	TPM2	22004	tropomyosin 2, beta
	ANGEL2	52477	angel homolog 2
	STARD9	668880	START domain containing 9
	KRT8	16691	keratin 8
	HFE2	69585	hemochromatosis type 2
	CTPS	51797	cytidine 5'-triphosphate synthase
	COL4A2	12827	collagen, type IV, alpha 2
	COL4A1	12826	collagen, type IV, alpha 1
	PNLDC1	240023	poly
	MYADM	50918	myeloid-associated differentiation marker
	CFD	11537	complement factor D
	CDCA3	14793	cell division cycle associated 3
	PEX11A	18631	peroxisomal biogenesis factor 11 alpha
	ILDR2	100039795	immunoglobulin-like domain containing receptor 2
	ADRB2	11555	adrenergic receptor, beta 2
	PLA2G6	53357	phospholipase A2, group VI
	NLN	75805	neurolysin

PITRM1	69617	pitrilysin metallepetidase 1
CLEC7A	56644	C-type lectin domain family 7, member a
UBD	24108	ubiquitin D
UAP1L1	227620	UDP-N-acteylglucosamine pyrophosphorylase 1-like 1
PSAP	19156	prosaposin
ATP6V1C1	66335	ATPase, H+ transporting, lysosomal V1 subunit C1
WWTR1	97064	WW domain containing transcription regulator 1
EGR2	13654	early growth response 2
VCAM1	22329	vascular cell adhesion molecule 1
MLKL	74568	mixed lineage kinase domain-like
PMM1	29858	phosphomannomutase 1
SMURF1	75788	SMAD specific E3 ubiquitin protein ligase 1
IDH2	269951	isocitrate dehydrogenase 2
TNFRSF10B	21933	tumor necrosis factor receptor superfamily, member 10b
RMDN3	67809	regulator of microtubule dynamics 3
SMARCA4	20586	SWI/SNF related, matrix associated, actin dependent regulator of chromatin, subfamily a, member 4
MFSD7C	217721	major facilitator superfamily domain containing 7C
RCAN1	54720	regulator of calcineurin 1
TMEM98	103743	transmembrane protein 98
P4HA2	18452	procollagen-proline, 2-oxoglutarate 4-dioxygenase
TMEM237	381259	transmembrane protein 237
CDK1	12534	cyclin-dependent kinase 1
MAGED2	80884	melanoma antigen, family D, 2
TGFBI	21810	transforming growth factor, beta induced
REEP6	70335	receptor accessory protein 6
GALK1	14635	galactokinase 1
LGALS3BP	19039	lectin, galactoside-binding, soluble, 3 binding protein
TOP2A	21973	topoisomerase
DET1	76375	de-etiolated homolog 1
CXCL9	17329	chemokine
SRXN1	76650	sulfiredoxin 1 homolog
HEXB	15212	hexosaminidase B
HEXA	15211	hexosaminidase A
KRT23	94179	keratin 23
PCSK6	18553	proprotein convertase subtilisin/kexin type 6
PHF11D	219132	PHD finger protein 11D
SMPD3	58994	sphingomyelin phosphodiesterase 3, neutral
ADAMTS2	216725	a disintegrin-like and metallopeptidase
CES2G	72361	carboxylesterase 2G

EAR2	13587	eosinophil-associated, ribonuclease A family, member 2
SKP1A	21402	S-phase kinase-associated protein 1A
ABCD1	11666	ATP-binding cassette, sub-family D
SREBF1	20787	sterol regulatory element binding transcription factor 1
MANBA	110173	mannosidase, beta A, lysosomal
SLC13A3	114644	solute carrier family 13
ANXA2	12306	annexin A2
RFC1	19687	replication factor C
PLEKHG5	269608	pleckstrin homology domain containing, family G
IFNGR2	15980	interferon gamma receptor 2
HEG1	77446	heart development protein with EGF-like domains 1
SLC39A11	69806	solute carrier family 39
PAWR	114774	PRKC, apoptosis, WT1, regulator
DUSP6	67603	dual specificity phosphatase 6
TGFBR2	21813	transforming growth factor, beta receptor II
DUSP22	105352	dual specificity phosphatase 22
PKM	18746	pyruvate kinase, muscle
TMEM8	60455	transmembrane protein 8
AEBP2	11569	AE binding protein 2
PITPNM2	19679	phosphatidylinositol transfer protein, membrane-associated 2
PLIN4	57435	perilipin 4
EXOC4	20336	exocyst complex component 4
MCM4	17217	minichromosome maintenance complex component 4
PPARG	19016	peroxisome proliferator activated receptor gamma
SLC25A10	27376	solute carrier family 25
MCM6	17219	minichromosome maintenance complex component 6
MICU2	68514	mitochondrial calcium uptake 2
TLR5	53791	toll-like receptor 5
MFGE8	17304	milk fat globule-EGF factor 8 protein
ALDOA	11674	aldolase A, fructose-bisphosphate
GAPDH	14433	glyceraldehyde-3-phosphate dehydrogenase
MGLL	23945	monoglyceride lipase
MCM2	17216	minichromosome maintenance complex component 2
CD63	12512	CD63 antigen
CEBPB	12608	CCAAT/enhancer binding protein
PDXK	216134	pyridoxal
PCX	18563	pyruvate carboxylase
NR1I3	12355	nuclear receptor subfamily 1, group I, member 3
DLST	78920	dihydrolipoamide S-succinyltransferase

	MAT2B	108645	methionine adenosyltransferase II, beta
	ACNAT2	209186	acyl-coenzyme A amino acid N-acyltransferase 2
	HSD17B10	15108	hydroxysteroid
	CLN6	76524	ceroid-lipofuscinosis, neuronal 6
	ADCY6	11512	adenylate cyclase 6
	TRAK2	70827	trafficking protein, kinesin binding 2
	ERMP1	226090	endoplasmic reticulum metallopeptidase 1
	HAUS8	76478	4HAUS augmin-like complex, subunit 8
	GPNMB	93695	glycoprotein
	MKNK2	17347	MAP kinase-interacting serine/threonine kinase 2
	ANO10	102566	anoctamin 10
	GBP2	14469	guanylate binding protein 2
	CBR3	109857	carbonyl reductase 3
	PAIP1	218693	polyadenylate binding protein-interacting protein 1
	GBP3	55932	guanylate binding protein 3
	MPP1	17524	membrane protein, palmitoylated
	NTRK2	18212	neurotrophic tyrosine kinase, receptor, type 2
	GDF15	23886	growth differentiation factor 15
	GSS	14854	glutathione synthetase
	GATB	229487	glutamyl-tRNA
	MYO9B	17925	myosin IXb
	FMO5	14263	flavin containing monooxygenase 5
	PFKL	18641	phosphofructokinase, liver, B-type
	GNPDA1	26384	glucosamine-6-phosphate deaminase 1
	ABHD17A	216169	abhydrolase domain containing 17A
	GSTA1	14857	glutathione S-transferase, alpha 1
	UBAP1	67123	ubiquitin-associated protein 1
	VIM	22352	vimentin
	GPR146	80290	G protein-coupled receptor 146
Down	USP6NL	98910	USP6 N-terminal like
	HSPB6	243912	heat shock protein, alpha-crystallin-related, B6
	KEG1	64697	kidney expressed gene 1
	RPLP0	11837	ribosomal protein, large, P0
	ATP2A2	11938	ATPase, Ca++ transporting, cardiac muscle, slow twitch 2
	IL1RAP	16180	interleukin 1 receptor accessory protein
	HAPLN4	330790	hyaluronan and proteoglycan link protein 4
	TXNDC15	69672	thioredoxin domain containing 15
	FTCD	14317	formiminotransferase cyclodeaminase
	TM7SF2	73166	transmembrane 7 superfamily member 2

ZFP160	224585	zinc finger protein 160
SPATA22	380709	spermatogenesis associated 22
KYNU	70789	kynureninase
DNMT3B	13436	DNA methyltransferase 3B
UNKL	74154	unkempt family like zinc finger
NCKAP5	210356	NCK-associated protein 5
PLEKHJ1	78670	pleckstrin homology domain containing, family J member 1
TLE4	21888	transducin-like enhancer of split 4
CAMK1D	227541	calcium/calmodulin-dependent protein kinase ID
ELOVL3	12686	elongation of very long chain fatty acids
PPP2R5D	21770	protein phosphatase 2, regulatory subunit B', delta
ALG12	223774	asparagine-linked glycosylation 12
MOGS	57377	mannosyl-oligosaccharide glucosidase
SOX12	20667	SRY
AVPR1A	54140	arginine vasopressin receptor 1A
PDIA6	71853	protein disulfide isomerase associated 6
CLPX	270166	caseinolytic mitochondrial matrix peptidase chaperone subunit
AGTR1A	11607	angiotensin II receptor, type 1a
WDR33	74320	WD repeat domain 33
CES3B	13909	carboxylesterase 3B
GLTPD2	216871	glycolipid transfer protein domain containing 2
ADGRF1	77596	adhesion G protein-coupled receptor F1
DOK3	27261	docking protein 3
PRKD3	75292	protein kinase D3
GFOD2	70575	glucose-fructose oxidoreductase domain containing 2
SLCO2A1	24059	solute carrier organic anion transporter family, member 2a1
TMEM218	66279	transmembrane protein 218
RBMS1	56878	RNA binding motif, single stranded interacting protein 1
ODF3B	70113	outer dense fiber of sperm tails 3B
NSMF	56876	NMDA receptor synaptonuclear signaling and neuronal migration factor
ACPP	56318	acid phosphatase, prostate
AHCY	269378	S-adenosylhomocysteine hydrolase
PNKP	59047	polynucleotide kinase 3'- phosphatase
TSHZ2	228911	teashirt zinc finger family member 2
GLT8D1	76485	glycosyltransferase 8 domain containing 1
ADH7	11529	alcohol dehydrogenase 7
EGFR	13649	epidermal growth factor receptor
RXRB	20182	retinoid X receptor beta
EFNB1	13641	ephrin B1

ADH4	26876	alcohol dehydrogenase 4
MYG1	60315	melanocyte proliferating gene 1
PORCN	53627	porcupine homolog
APOM	55938	apolipoprotein M
FGD6	13998	FYVE, RhoGEF and PH domain containing 6
RIC8B	237422	RIC8 guanine nucleotide exchange factor B
RDH11	17252	retinol dehydrogenase 11
CBS	12411	cystathionine beta-synthase
PMVK	68603	phosphomevalonate kinase
ASPDH	68352	aspartate dehydrogenase domain containing
RPS3	27050	ribosomal protein S3
NPHP3	74025	nephronophthisis 3
HACL1	56794	2-hydroxyacyl-CoA lyase 1
SRGAP3	259302	SLIT-ROBO Rho GTPase activating protein 3
CCL25	20300	chemokine
HEXIM1	192231	hexamethylene bis-acetamide inducible 1
ZFP715	69930	zinc finger protein 715
NR1H2	22260	nuclear receptor subfamily 1, group H, member 2
SERPINA3K	20714	serine
MCC	328949	mutated in colorectal cancers
GNMT	14711	glycine N-methyltransferase
FBXL12	30843	F-box and leucine-rich repeat protein 12
NUDT7	67528	nudix
CYP2C67	545288	cytochrome P450, family 2, subfamily c, polypeptide 67
FABP5	16592	fatty acid binding protein 5, epidermal
CNOT1	234594	CCR4-NOT transcription complex, subunit 1
EIF3I	54709	eukaryotic translation initiation factor 3, subunit I
RPL27A	26451	ribosomal protein L27A
CTH	107869	cystathionase
CAPRIN1	53872	cell cycle associated protein 1
CWF19L1	72502	CWF19-like 1, cell cycle control
PXMP4	59038	peroxisomal membrane protein 4
RBAK	57782	RB-associated KRAB zinc finger
FGFR4	14186	fibroblast growth factor receptor 4
GSTM7	68312	glutathione S-transferase, mu 7
GM10319	381806	murinoglobulin pseudogene
ZFP672	319475	zinc finger protein 672

Supplementary Table 9. Clinical background of liver tissue donors

Sample ID	Age	Sex	Pathology diagnosis	Type
NORMAL_I11	47	M	Liver tissue	Normal
NORMAL_I12	27	M	Liver tissue (sparse)	Normal
NORMAL_J1	35	M	Liver tissue	Normal
NORMAL_J2	38	M	Liver tissue	Normal
NORMAL_J3	21	F	Liver tissue	Normal
NORMAL_J4	45	M	Liver tissue	Normal
NORMAL_J5	43	M	Liver tissue	Normal
NORMAL_J6	50	F	Liver tissue	Normal
NORMAL_J7	16	M	Liver tissue	Normal
NORMAL_J8	40	M	Liver tissue	Normal
NORMAL_J9	27	F	Liver tissue	Normal
NORMAL_J10	23	M	Liver tissue	Normal
NORMAL_J11	45	M	Liver tissue	Normal
NORMAL_J12	36	M	Liver tissue	Normal
NAFLD_D5	54	M	Liver tissue with fatty degeneration	Degeneration
NAFLD_D6	32	M	Liver tissue with fatty degeneration	Degeneration
NAFLD_D8	45	F	Liver tissue with fatty degeneration	Degeneration
NAFLD_D9	74	F	Liver tissue with fatty degeneration	Degeneration
NAFLD_D10	69	M	Liver tissue with fatty degeneration	Degeneration
NAFLD_D11	38	M	Liver tissue with fatty degeneration	Degeneration
NAFLD_D12	36	M	Chronic active hepatitis with fatty degeneration	Degeneration
NAFLD_E2	42	M	Liver tissue with fatty degeneration	Degeneration
NAFLD_E4	50	M	Liver tissue with fatty degeneration	Degeneration
NAFLD_E5	54	F	Liver tissue with fatty degeneration	Degeneration
NAFLD_E6	37	M	Liver tissue with fatty degeneration	Degeneration
NAFLD_E7	60	M	Liver tissue with fatty degeneration	Degeneration
NAFLD_E8	54	M	Liver tissue with fatty degeneration	Degeneration

Supplementary Table 10. Immunohistochemistry readings

Sample ID	GBP2	CMIP
NAFLD_D5	2	1
NAFLD_D6	1	1
NAFLD_D8	1	1
NAFLD_D9	0	1
NAFLD_D10	2	1
NAFLD_D11	2	0
NAFLD_D12	2	focal 2
NAFLD_E2	1	2
NAFLD_E4	2	focal 1
NAFLD_E5	0	1
NAFLD_E6	0	2
NAFLD_E7	1	2
NAFLD_E8	2	2

Supplementary Figures

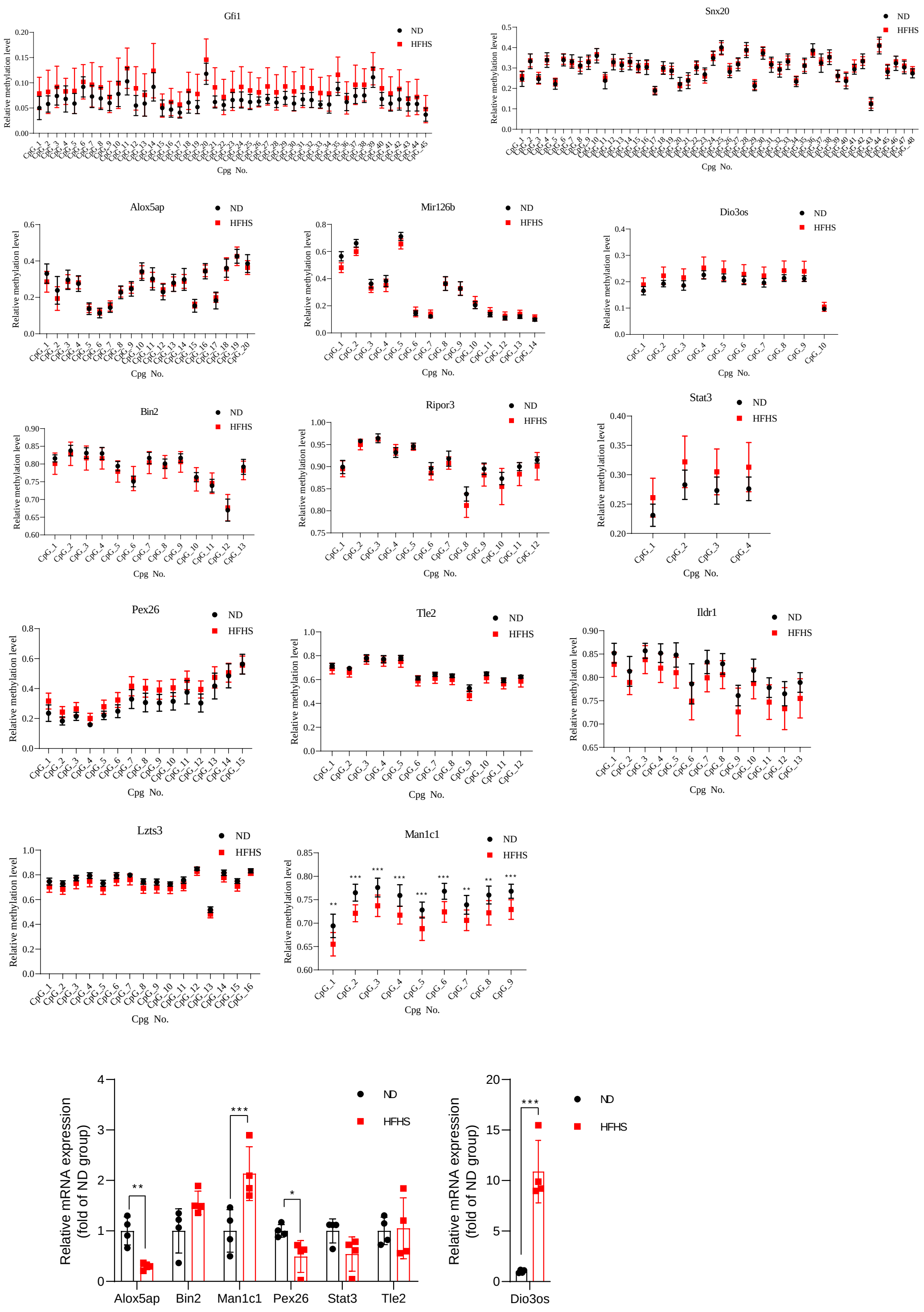

Supplementary Fig. 1. DNA methylation level and mRNA expression in mouse liver tissues. **(a)** Targeted DNA methylation analysis of 13 candidate genes. The relative DNA methylation levels of CpG sites located in each gene were measured by BSAS (n=9-10/each group). **(b)** Gene expression analysis of *Alox5ap*, *Bin2*, *Dio3os*, *Man1c1*, *Pex26*, *Stat3*, and *Tle2* by qRT-PCR. The values presented are the means ± SD (n=5-7/each group). * p < 0.05, ** p < 0.01, and *** p < 0.001. ND, normal diet; HFHS, High-fat and high-sucrose diet.

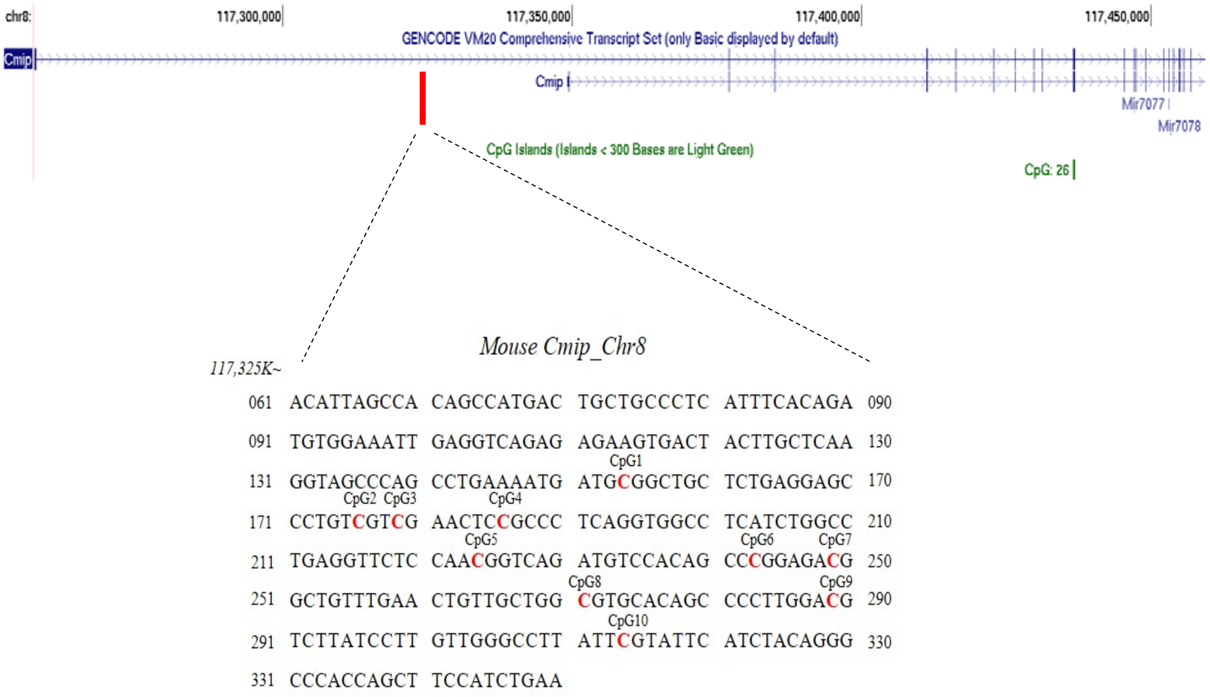

Supplementary Fig. 2. DNA sequences of the DMR region in Cmip intron 1. 10 CpG sites in the region are marked in red.

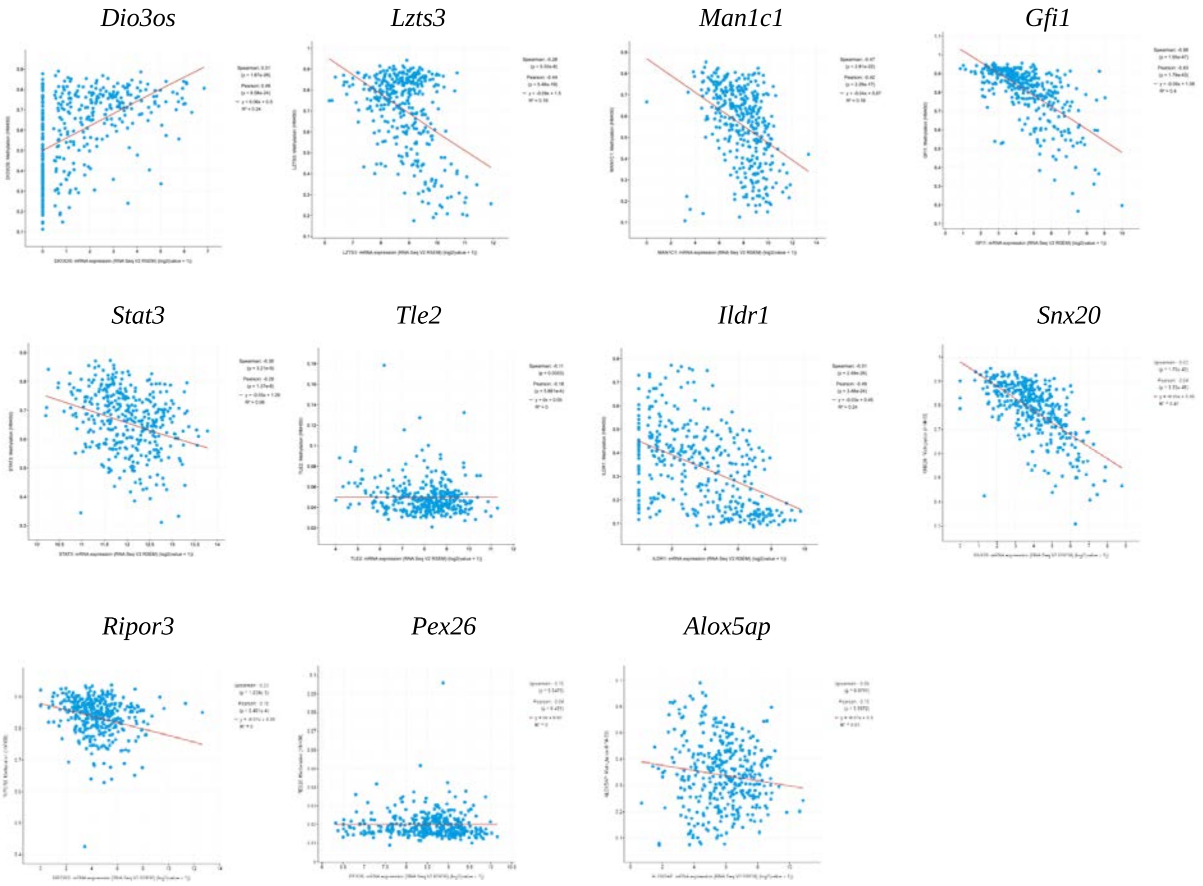

Supplementary Fig. 3. Correlation analysis of gene expression and DNA methylation levels using human tissue transcriptome and DNA methylation data (n=373) from publicly available databases. Data were analyzed using Pearson's correlation coefficient (R). Genes with a positive correlation are indicated in red, and genes with a negative correlation are indicated in blue. x = mRNA expression level, y = DNA methylation level.

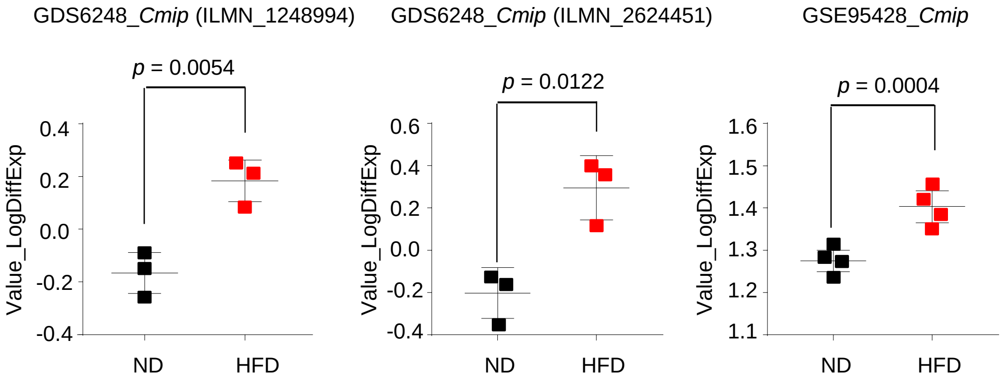

Supplementary Fig. 4. Data from publicly available datasets on Cmip mRNA expression levels in liver tissues of mice supplemented with a high-fat diet (HFD) or normal diet (ND). Cmip mRNA expression was higher in the HFD-induced non-alcoholic fatty liver disease (NAFLD) model in vivo. GDS6248, n = 3/group; GSE95428, n = 4/group. Quantification of Cmip mRNA levels by microarray (two probes; ILMN_1248994 and ILMN_2624451) in the GDS6248 dataset (left and middle panels) and by RNA-seq in GDS95428 (right panel) reveals that Cmip mRNA levels were significantly higher in HFD groups than in ND groups ($p < 0.05$).

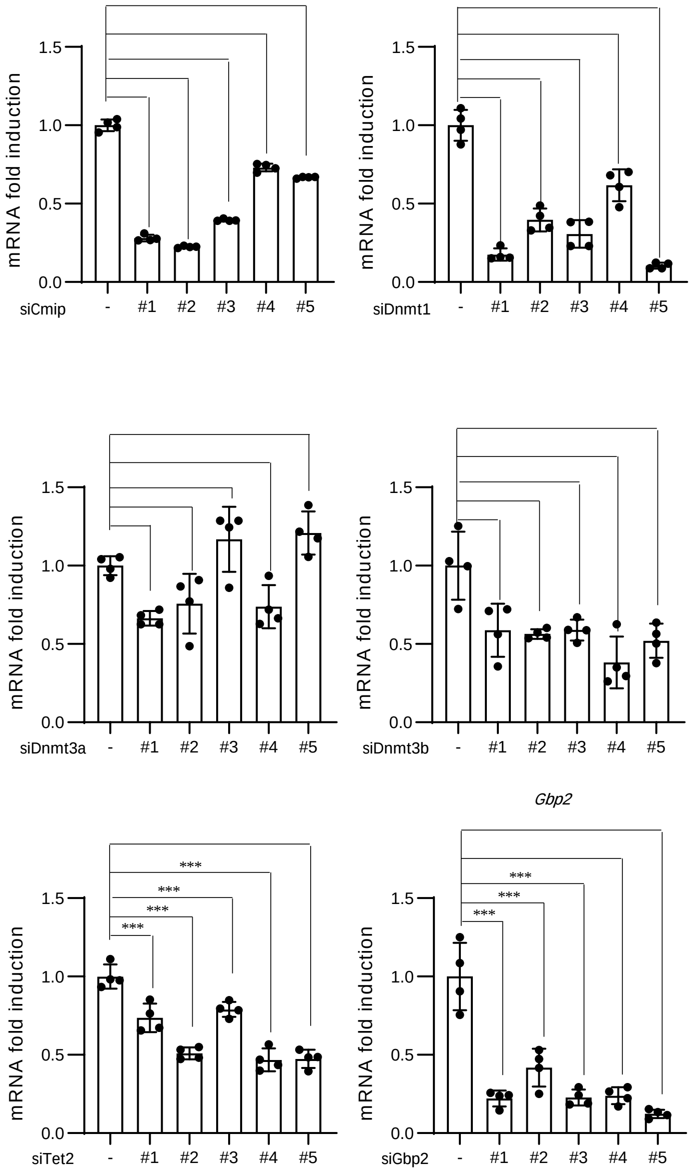

Supplementary Fig. 5. siRNAs were transiently transfected into AML-12 cells. After 48 h, cells were harvested, lysed, and total RNA were extracted. mRNA expression of the genes was measured by qRT-PCR. The top two siRNAs showing the greatest knockdown effect were used for further knockdown experiments. The values presented are the means ± standard deviation of three independent experiment. *$p < 0.05$, **$p < 0.01$, ***$p < 0.001$, and ****$p < 0.0001$.

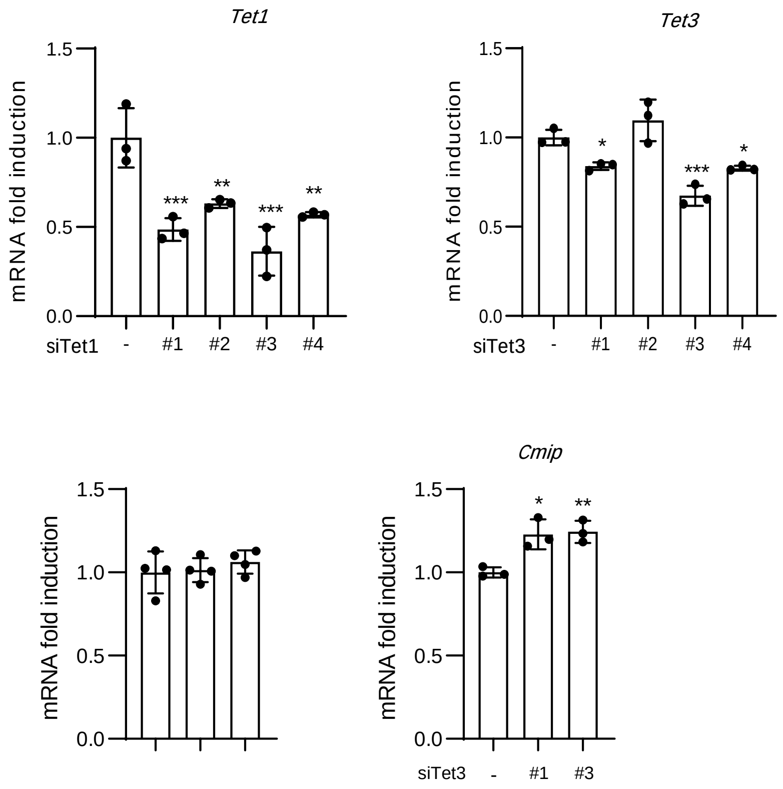

Supplementary Fig. 6. Knockdown of either Tet1 or Tet3 did not significantly affect in Cmip mRNA expression. **(a)** Either Tet1 or Tet3 siRNAs was transiently transfected into AML-12 cells. After 48 h, cells were harvested, lysed, and then total RNA were extracted. mRNA expression of the genes was measured by qRT-PCR. The top two siRNAs showing highest knockdown effect were used for the knockdown experiments. The values presented are the means ± standard deviation (SD) of three independent experiment. $*p < 0.05$, $**p < 0.01$, and $***p < 0.001$. **(b)** The indicated siRNAs were transfected in AML-12 cells. After 48 h, Cmip mRNA expression was measured using qRT-PCR. The values presented are the means ± SD of three independent experiment. $*p < 0.05$, $**p < 0.01$, and $***p < 0.001$.

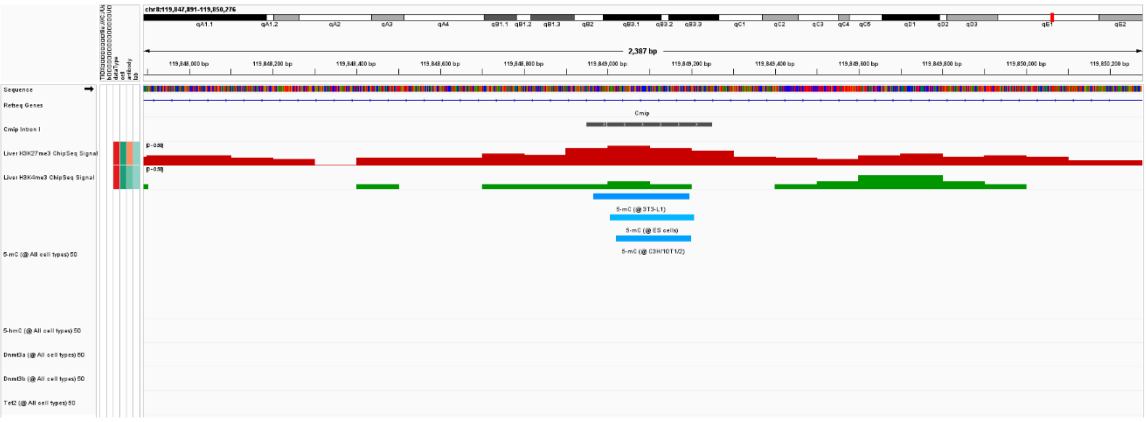

Supplementary Fig. 7. ChIP-seq genome browser tracks for Cmip intron 1 location, murine liver H3K27me3 and cellular 5-methylcytosine (5-mC), 5-hydroxymethylcytosine (5-hmC), Dnmt3a, Dnmt3b, and Tet2 signals. Integrative Genomics Viewer (IGV) software (Ver. 2.12.3); mouse (NCBI37/mm9) genome DB. ChIP-seq data from ChIP-Atlas (Oki, S; Ohta, T (2015): ChIP-Atlas. https://chip-atlas.org)

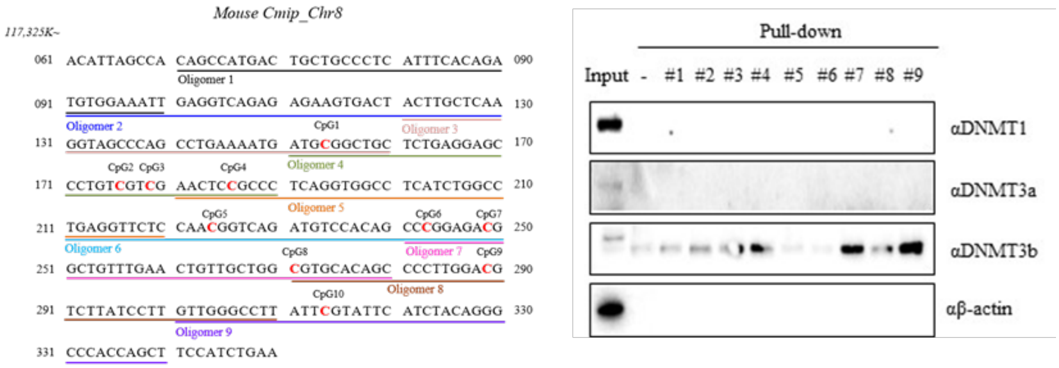

Supplementary Fig. 8. Dnmt3b directly binds to *Cmip* introgenic region in AML-12 cells. Oligonucleotide pull-down assay was performed by reacting the oligonucleotides of Cmip intronic region and AML-12 cell lysates.

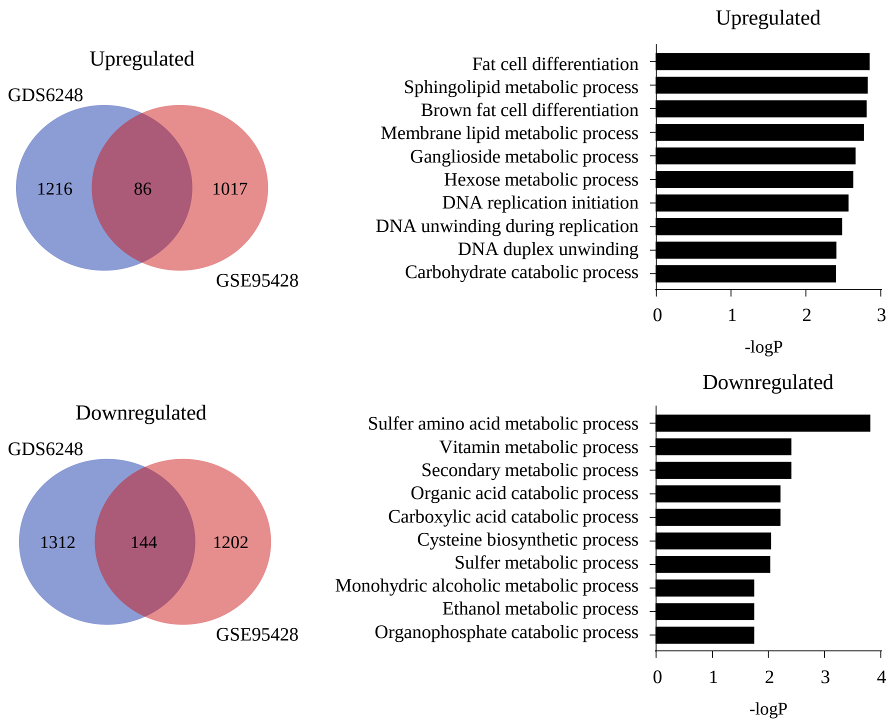

Supplementary Fig. 9. GO enrichment analysis

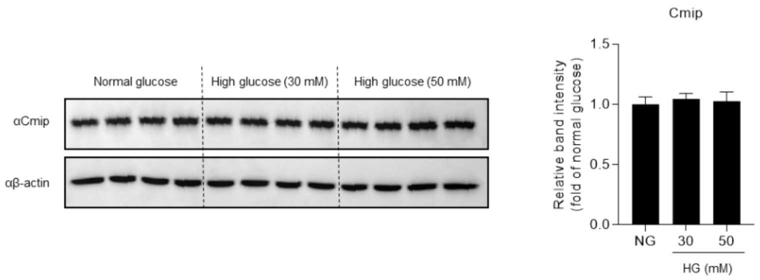

Supplementary Fig. 10. The effect of high (30 or 50 mM) or normal (5.5 mM) glucose level on Cmip expression in AML-12 cells. Normal or high glucose was treated to the cells for 24 h, then the proteins were detected by western blotting. The cells were immunoblotted with the indicated antibodies (left panel). The intensities of protein bands obtained from the western blot assays were quantified using ImageJ (right panel) and normalized with respect to that of β-actin. Relative fold intensity was calculated by the sum of normalized intensities from both β-actin and Cmip.

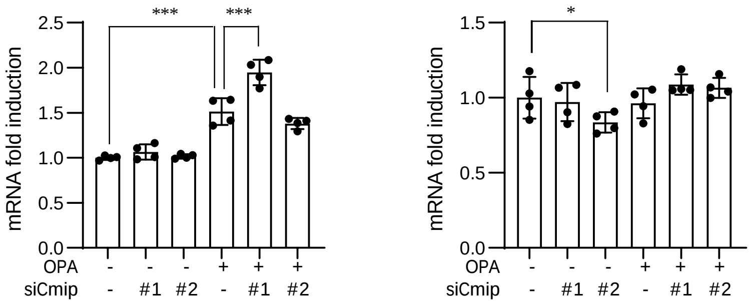

Supplementary Fig. 11. Knockdown of Cimp did not significantly affect in Cebpβ or Pex11α mRNA expression. Cmip siRNA was transiently transfected into AML-12 cells. After 48 h, cells were harvested, lysed, and total RNA was extracted. mRNA expression of the genes was measured by qRT-PCR. The values presented are the means ± standard deviation of three independent experiment. *p < 0.05, **p < 0.01, and ***p < 0.001.

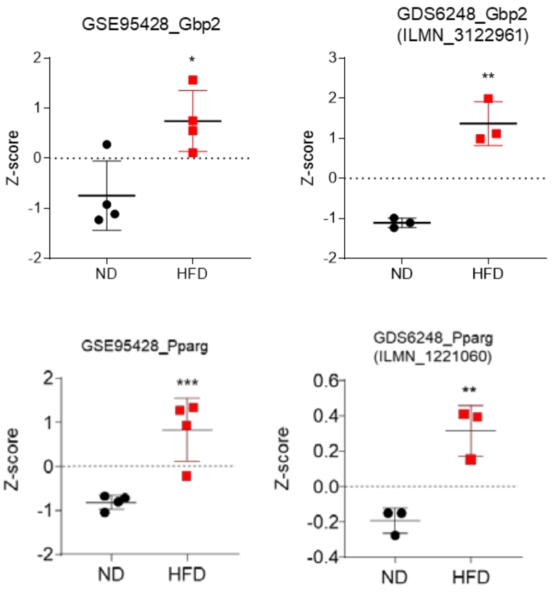

Supplementary Fig. 12. Data from publicly available datasets on Cmip mRNA expression levels in liver tissues of mice supplemented with a high-fat diet (HFD) or normal diet (ND). *Gbp2* or *Pparg* mRNA expression was higher in the HFD-induced non-alcoholic fatty liver disease (NAFLD) model *in vivo*. GDS6248, n = 3/group; GSE95428, n = 4/group. Quantification of *Gbp2* or *Pparg* mRNA levels by microarray in the GDS6248 dataset (left panel) and by RNA-seq in GDS95428 (right panel) reveals that *Gbp2* or *Pparg* mRNA levels were significantly higher in HFD groups than in ND groups. *, $p < 0.05$; **, $p < 0.01$; ***, $p < 0.001$ (compared to ND group).

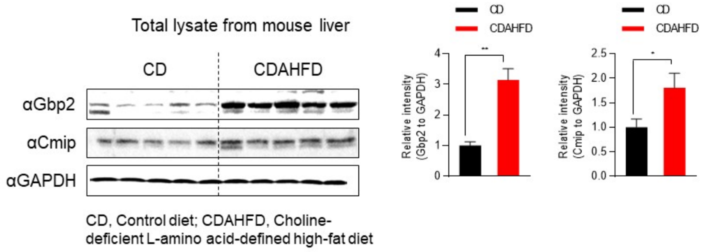

Supplementary Fig. 13. The level of *Cmip* expression in liver tissues from either CD or CDAHFD-fed mice as detected by western blotting. Liver lysates were immunoblotted with the indicated antibodies (left panel). The intensities of protein bands obtained from the western blot assays were quantified using ImageJ (right panel) and normalized with respect to that of β-actin. Relative fold intensity was calculated by the sum of normalized intensities from both β-actin and Cmip. *$p < 0.05$, ** $p < 0.01$.
